# Parabacteroides produces acetate to alleviate heparanase-exacerbated acute pancreatitis through reducing neutrophil infiltration

**DOI:** 10.1186/s40168-021-01065-2

**Published:** 2021-05-20

**Authors:** Yuanyuan Lei, Li Tang, Shuang Liu, Shiping Hu, Lingyi Wu, Yaojiang Liu, Min Yang, Shengjie Huang, Xuefeng Tang, Tao Tang, Xiaoyan Zhao, Israel Vlodavsky, Shuo Zeng, Bo Tang, Shiming Yang

**Affiliations:** 1 Department of Gastroenterology, Third Military Medical University Second Affiliated Hospital, Chongqing, 400037 China; 2 Department of Gastroenterology, The 983rd Hospital of Chinese PLA Joint Logistics Support Force, Tianjin, 300142 China; 3 Department of Gastroenterology, Affiliated Hospital of North Sichuan Medical College, Nanchong, 637000 China; 4 Department of Gastroenterology, Chongqing Medical University Affiliated Second Hospital, Chongqing, 400010 China; 5 Department of Pathology, Third Military Medical University Second Affiliated Hospital, Chongqing, 400037 China; 6 Laboratory Department, Third Military Medical University Second Affiliated Hospital, Chongqing, 400037 China; 7 Technion Integrated Cancer Center (TICC), Rappaport Faculty of Medicine, Technion-Israel Institute of Technology, 31096 Haifa, Israel

## Abstract

**Background:**

The endoglycosidase heparanase which degrades heparan sulfate proteoglycans, exerts a pro-inflammatory mediator in various inflammatory disorders. However, the function and underlying mechanism of heparanase in acute pancreatitis remain poorly understood. Here, we investigated the interplay between heparanase and the gut microbiota in the development of acute pancreatitis.

**Methods:**

Acute pancreatitis was induced in wild-type and heparanase-transgenic mice by administration of caerulein. The differences in gut microbiota were analyzed by 16S ribosomal RNA sequencing. Antibiotic cocktail experiment, fecal microbiota transplantation, and cohousing experiments were used to assess the role of gut microbiota.

**Results:**

As compared with wild-type mice, acute pancreatitis was exacerbated in heparanase-transgenic mice. Moreover, the gut microbiota differed between heparanase-transgenic and wild-type mice. Heparanase exacerbated acute pancreatitis in a gut microbiota-dependent manner. Specially, the commensal *Parabacteroid*es contributed most to distinguish the differences between wild-type and heparanase-transgenic mice. Administration of *Parabacteroides* alleviated acute pancreatitis in wild-type and heparanase-transgenic mice. In addition, *Parabacteroides* produced acetate to alleviate heparanase-exacerbated acute pancreatitis through reducing neutrophil infiltration.

**Conclusions:**

The gut–pancreas axis played an important role in the development of acute pancreatitis and the acetate produced by *Parabacteroides* may be beneficial for acute pancreatitis treatment.

Video abstract

**Supplementary Information:**

The online version contains supplementary material available at 10.1186/s40168-021-01065-2.

## Introduction

Acute pancreatitis (AP) is a common disease that manifests as acute abdominal pain and involves pancreatic enzyme activation and pancreatic “self-digestion”. Globally, AP affects a large proportion of the population, and the incidence is more than 34 cases per 100,000 general population per year [1]. Although most AP are mild, some patients with AP experience rapid aggravation, and the mortality rate is as high as 20–30% [2], resulting in increasing health care expenditures and long-term hospitalization [3, 4]. Therefore, there is an urgent need to prevent AP exacerbation.

The endoglycosidase heparanase (Hpa) degrades heparan sulfate (HS) proteoglycans. Hpa has been identified as a pro-inflammatory mediator in various inflammatory disorders, such as rheumatoid arthritis [5], Barrett’s esophagus [6], Crohn’s disease [7], ulcerative colitis [8], and sepsis [9]. Hpa overexpression is observed in human chronic pancreatitis [10]. In AP, Hpa inhibitor PG545 alleviates pancreatic inflammation in mice [11]. Therefore, Hpa emerges as a potential novel target in AP. However, the specific mechanism by which Hpa promotes AP remains unclear.

A new research filed suggests that the crosstalk between pancreas and gut microbiota is involved in the development of pancreas disorders [12], including pancreatic cancer [13], diabetes [14], autoimmune pancreatitis [15], and chronic pancreatitis [15, 16]. Specially, gut microbiota differs between AP patients and healthy volunteers [17]. Moreover, gut microbiota differs among AP patients with different severity grades [18, 19]. In addition, depleting the gut microbiota using an antibiotic cocktail (ABX) alleviates AP in mice [20]. These studies indicate that AP might be modulated by the gut microbiota. However, which commensal is the most important biomarker and the mechanism by which the gut microbiota regulates the development of AP remain unclear.

Interestingly, emerging evidences indicate that host genetics shape part of the gut microbiota to participate in the regulation of disease phenotypes [21]. Microbiota features associated with genetic variations include the gut microbiota composition, diversity, structure, metabolic changes, and bacterial colonization patterns [22, 23]. Report demonstrates that TLR5-deficient mice harbor a gut microbiota which generates more cecal oleate and substrates for hepatic lipogenesis [24]. The interplay between the gut microbiota and host gene NLRP3 affects the development of AP [25]. Given that host genes play an important role in gut microbiota, we aimed to assess the interaction between Hpa and the gut microbiota in a mouse model of AP.

In this study, we observed that AP was exacerbated in transgenic mice overexpressing hpa (Hpa-Tg mice) as compared with littermate controls. The results of 16S ribosomal RNA (rRNA) sequencing, ABX, fecal microbiota transplantation (FMT), and cohousing experiments reported that a changed gut microbiota was responsible for Hpa-exacerbated AP. Moreover, the underlying mechanism of the gut–pancreas axis in Hpa-exacerbated AP was also investigated.

## Results

### Acute pancreatitis was exacerbated in Hpa-Tg mice

**Fig. 1:**
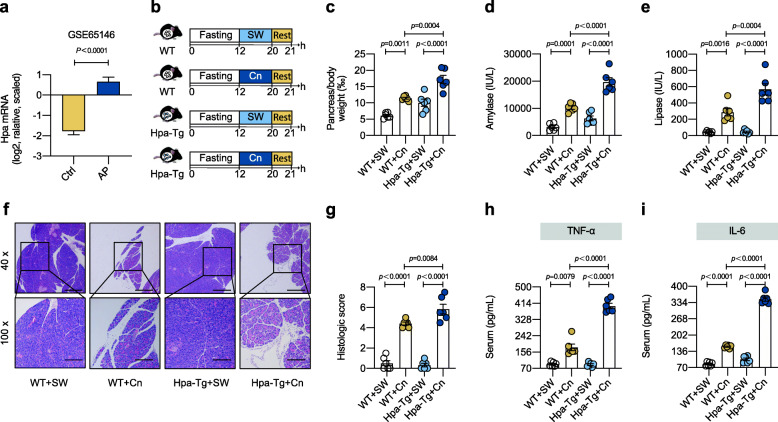
Acute pancreatitis was exacerbated in Hpa-Tg mice. **a** In WT mice, Hpa mRNA expression in control (*n* = 5) and AP (*n* = 9) was compared. Data were from GEO database GSE65146. **b** AP induction experimental design. WT and Hpa-Tg littermates were fasted for 12 h and injected with either Cn or SW. Mice were sacrificed 1 h after the last injection. **c** Pancreas (g)/body weight (g) × 1‰. **d** Serum amylase. **e** Serum lipase. **f** Representative images of pancreatic H&E staining. **g** Histologic score. **h** Serum TNF-α measured by ELISA. **i** Serum IL-6 measured by ELISA. **b–i** *n* = 6 individuals/group. Data were expressed as mean ± SEM. Differences of data between two groups were assessed by unpaired, two-tailed *t* test (**a**). Differences of data between multiple groups were assessed by ordinary one-way ANOVA (**b–i**). Exact *p* levels were all provided. Scale bars, 500 and 200 μm, respectively. *AP*, acute pancreatitis; *Cn*, caerulein; *GEO*, Gene Expression Omnibus database; *Hpa-Tg*, heparanase-transgenic; *SW*, sterile water; *WT*, wild type

First, we found that compared with the control mice, the overall Hpa mRNA level was significantly higher in AP mice (Fig. 1a). Then we used Hpa-Tg mice to further investigate the role of Hpa in AP (identification of the overexpressed genotype and confirmation of mouse and human Hpa encoding gene expressions across all experimental conditions were shown in Figure S1). We compared wild-type (WT) and Hpa-Tg mice in a caerulein (Cn)-induced AP model (Fig. 1b). Notably, Hpa-Tg mice exhibited increased pancreas/body weight (‰), raised serum amylase, raised serum lipase, and higher histologic score as compared with WT mice (Fig. 1c–g). Additionally, Hpa-Tg mice displayed higher serum pro-inflammatory cytokines TNF-α and IL-6 (Fig. 1h, i). These data indicated that AP was significantly exacerbated in Hpa-Tg mice.

### The gut microbiota differed between Hpa-Tg mice and WT mice

**Fig. 2:**
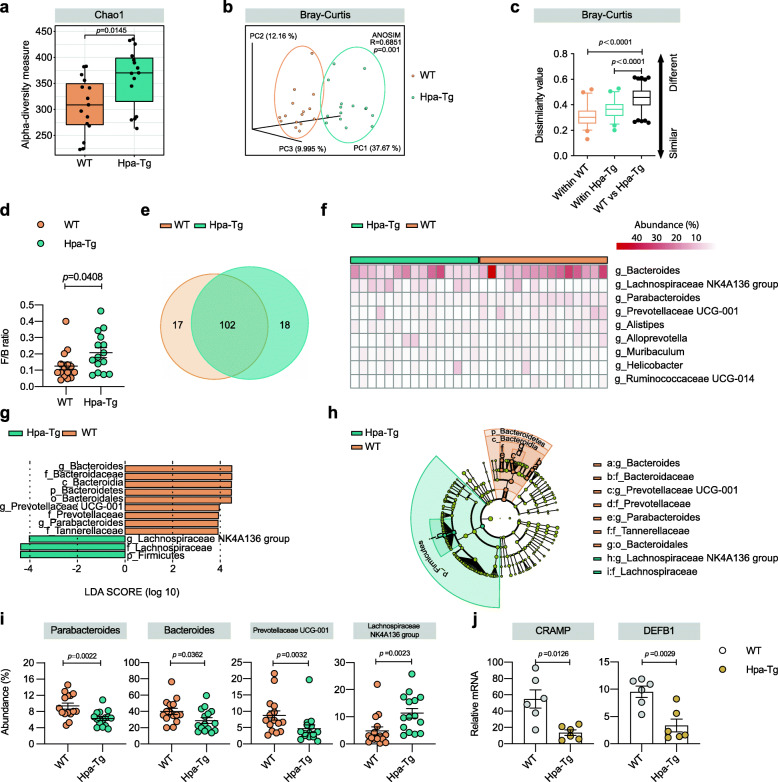
The gut microbiota differed between Hpa-Tg mice and WT mice. **a–i** 16S rRNA sequencing analysis in fecal bacterial DNA from healthy WT and Hpa-Tg littermates was performed. *n* = 15 individuals/group. **a** Alpha diversity (based on Chao1). **b** PCoA of beta diversity using Bray-Curtis metric distance. **c** Quantification of dissimilarity values based on (**b**), presented as dissimilarity values (first (box bottom), third (box top) quartiles, the median (line inside box) and 2.5 interquartile range (line ends)). **d** The ratio of *F*/*B* of WT and Hpa-Tg mice. **e** In genus level, Venn diagram of the ASVs in WT and Hpa-Tg mice. **f** Heatmap of selected differentially abundant features between WT and Hpa-Tg mice. **g** and **h** Cladograms generated by LEfSe indicating differences in the bacterial taxa between WT and Hpa-Tg mice. LDA score for the bacterial taxa differentially abundant between WT and Hpa-Tg mice (LDA > 3.5). Orange bars indicate taxa enrichment in WT mice, and green bars indicate taxa enrichment in Hpa-Tg mice. **i** The abundance of the genera *Parabacteroides*, *Bacteroides*, *Prevotellaceae UCG-001*, and *Lachnospiraceae NK4A 136 group* between WT and Hpa-Tg mice. **j** CRAMP and DEFB1 mRNA in intestinal tissue of healthy WT and Hpa-Tg mice. *n* = 6 individuals/group. Data were expressed as mean ± SEM. Differences of data between two groups were assessed by unpaired, two-tailed *t* test or Mann-Whitney test depending on the sample distribution. For (**b** and **c**)**,** differences of data were assessed by ANOSIM test. Exact *p* levels were all provided. *ANOSIM*, analysis of similarities; *ASVs*, amplicon sequence variants; *CRAMP*, cathelicidin-related antimicrobial peptide; *DEFB1*, β-defensin 1; *F/B, Firmicutes*/*Bacteroidetes*; *Hpa-Tg*, heparanase-transgenic; *LDA*, linear discriminant analysis; *LEfSe*, linear discriminant analysis effect size; *PCoA*, principal coordinate analysis; *rRNA*, ribosomal RNA; *WT*, wild type

Then we performed 16S rRNA sequencing analysis to investigate the differences in gut microbiota derived from WT and Hpa-Tg genotypes. We measured alpha diversity using Chao1, ACE, Observed features, Fisher alpha, Shannon, and Faith pd (Fig. 2a and Figure S2a). Most of them manifested similar tendencies that Hpa-Tg mice harbored a microbiota with a slightly higher alpha diversity as compared with WT mice (*p* = 0.0145, *p* = 0.0142, *p* = 0.0136, *p* = 0.0132, *p* = 0.1485, and *p* = 0.0555 for each index). Then beta diversity analysis of principal coordinates analysis (PCoA) was performed using Bray-Curtis, Jaccard, Generalized Unifrac and Weighted Unifrac metric distance (Fig. 2b and Figure S2b). The result showed that the gut microbiota from different genotypes were largely separated (*p* = 0.001, *p* = 0.001, *p* = 0.002, and *p* = 0.001 for each analysis), suggesting that these communities were distinct in terms of their compositional structure. Then, we quantified the dissimilarity of bacterial communities among the groups. The results indicated that the microbiota difference between WT and Hpa-Tg mice was significantly greater than the difference between mice within each genotype (Fig. 2c and Figure S2c, calculated from Fig. 2b and Figure S2b). Next, we assessed the landscape of the gut microbiota in all available samples. At the phylum level, *Bacteroidetes* were the most predominant phyla, accounting for 86.79% and 80.23% of the gut microbiota in WT and Hpa-Tg mice, respectively (*p* = 0.0235). *Firmicutes* were the second most predominant phyla in both groups at 10.36% and 15.72%, respectively (*p* = 0.0367) (Figure S2d). Consistently, Hpa-Tg mice exhibited a significantly increased *Firmicutes*/*Bacteroidetes* (*F/B*) ratio (*p =* 0.0408) (Fig. 2d). The taxonomic composition of WT and Hpa-Tg mice was also compared at the class/order/family/genus level (Figure S2e-h). Overexpression of Hpa significantly altered gut microbiota taxonomic composition. At the genus level, Venn diagrams revealed 17 unique amplicon sequence variants (ASVs) in WT mice and 18 unique ASVs in Hpa-Tg mice. In total, 102 ASVs were shared by both groups (Fig. 2e). Heatmap showed the selected most differentially abundant features at the genus level between WT and Hpa-Tg mice (Fig. 2f).

Moreover, to detect the differences in the predominance of bacterial communities between these two groups, we performed high-dimensional class comparisons using the linear discriminant analysis (LDA) of effect size (LEfSe) (LDA score > 3.5). The genera *Bacteroides* (40.04% vs 28.69%, respectively; *p* = 0.0362), *Parabacteroides* (9.281% vs 6.239%, respectively; *p* = 0.0022) and *Prevotellaceae UCG-001* (8.708% vs 4.684%, respectively; *p* = 0.0032) exhibited relatively high abundance in WT mice, whereas the genus *Lachnospiraceae NK4A136 group* (4.810% vs 11.32%, respectively; *p* = 0.0023) was more abundant in Hpa-Tg mice (Fig. 2g, h). The abundance of these genera from WT and Hpa-Tg mice was shown in Fig. 2i. Furthermore, the cathelicidin-related antimicrobial peptide (CRAMP) and β-defensin 1 (DEFB1) were less abundant in Hpa-Tg mice compared with WT mice (Fig. 2j), which is consistent with previous work demonstrating that aggravated inflammation decreased the expression of antimicrobial peptides in AP, contributing to the gut microbiota changes [26]. Collectively, these data demonstrate that the gut microbiota differed between Hpa-Tg mice and WT mice.

### Hpa exacerbated AP in a gut microbiota-dependent manner

To determine whether the AP phenotype of Hpa-Tg mice depended on gut microbiota, we pretreated WT and Hpa-Tg mice with ABX for 5 days prior to Cn injection (Figure S3a). Surprisingly, ABX WT and ABX Hpa-Tg mice exhibited indistinguishable pancreas/body weight (‰), serum amylase, serum lipase, histology feature, serum TNF-α, and serum IL-6 levels (Figure S3b-h). Moreover, 16S rRNA sequencing was performed before AP induction to characterize the microbiome composition. Consistent with the ABX data, the gut microbiota composition of ABX WT mice was similar to that of ABX Hpa-Tg mice, as evidenced by beta diversity analysis of PCoA (Figure S4). These results indicated that exacerbated AP in Hpa-Tg mice depended on gut microbiota.

**Fig. 3:**
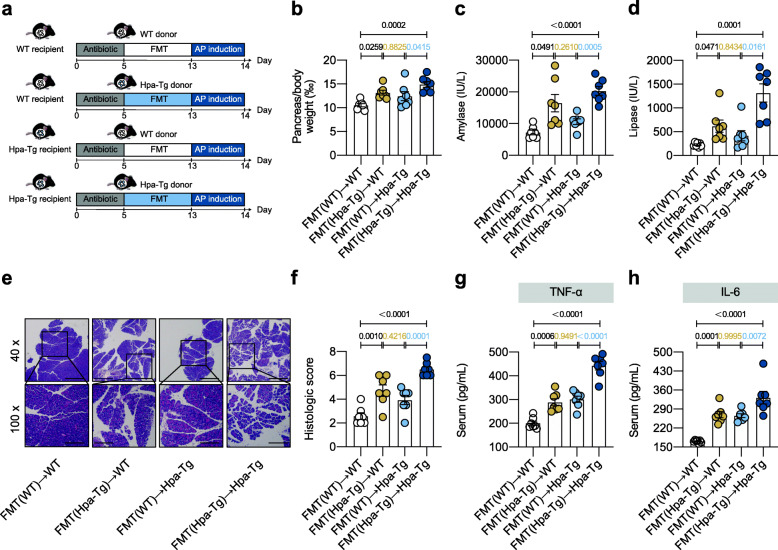
Transferring microbiota from Hpa-Tg mice exacerbated acute pancreatitis. **a** FMT experimental design. WT and Hpa-Tg littermates were put on a course of intragastrically antibiotic cocktail administration for 5 days for gut microbiota depletion prior to FMT and gavaged with the fecal contents of either WT or Hpa-Tg donor mice for 8 days. After FMT, 16S rRNA sequencing analysis and AP induction were performed. **b** Pancreas (g)/body weight (g) × 1‰. **c** Serum amylase. **d** Serum lipase. **e** Representative images of pancreatic H&E staining. **f** Histologic score. **g** Serum TNF-α measured by ELISA. **h** Serum IL-6 measured by ELISA. **a–h** *n* = 7 individuals/group. Data were expressed as mean ± SEM. Differences of data were assessed by ordinary one-way ANOVA or Kruskal-Wallis test depending on the sample distribution. Exact *p* levels were all provided. Scale bars, 500 and 200 μm, respectively. *AP*, acute pancreatitis; *FMT*, fecal microbiota transplantation; *Hpa-Tg*, heparanase-transgenic; *rRNA*, ribosomal RNA; *WT*, wild type

To further investigate the causality between Hpa-exacerbated AP and the altered gut microbiota, we conducted FMT experiment in which gut microbiota-depleted WT or Hpa-Tg mice (WT recipients or Hpa-Tg recipients) were reconstituted with the gut microbiota of WT or Hpa-Tg donor mice (Fig. 3a). The FMT (WT) → WT group resulted in significantly reduced pancreas/body weight (‰), serum amylase, serum lipase, histologic score, serum TNF-α,, and serum IL-6 levels as compared with the FMT(Hpa-Tg) → Hpa-Tg group. However, FMT(Hpa-Tg)→WT group and FMT(WT) → Hpa-Tg group exhibited similar AP phenotype (Fig. 3b–h). Consistent with the phenotype, 16S rRNA sequencing analysis showed that FMT(WT) → WT group displayed a distinct microbiota feature compared with FMT(Hpa-Tg) → Hpa-Tg group; FMT(Hpa-Tg) → WT group and FMT(WT) → Hpa-Tg group exhibited similar microbiota structure based on beta diversity analysis (Figure S5). These findings indicate that the gut microbiota shaped by the Hpa-Tg genotype contributed to the exacerbated AP.

**Fig. 4:**
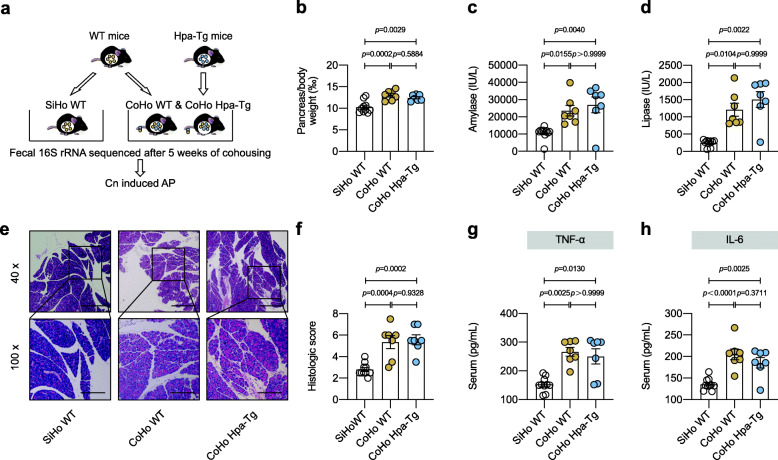
CoHo WT and CoHo Hpa-Tg mice displayed indistinguishable acute pancreatitis phenotype. **a** Cohousing experiment design. WT and Hpa-Tg littermates were divided to be either housed singly (SiHo WT) or cohoused (CoHo WT and CoHo Hpa-Tg mice at 1:1 ratio) for 5 weeks. After cohousing, 16S rRNA sequencing analysis and AP induction were performed. **b** Pancreas (g)/body weight (g) × 1‰. **c** Serum amylase. **d** Serum lipase. **e** Representative images of pancreatic H&E staining. **f** Histologic score. **g** Serum TNF-α measured by ELISA. **h** Serum IL-6 measured by ELISA. **a–h** *n* = 10/7/7 individuals in SiHo WT/ CoHo WT/ CoHo Hpa-Tg, respectively. Data were expressed as mean ± SEM. Differences of data were assessed by ordinary one-way ANOVA or Kruskal-Wallis test depending on the sample distribution. Exact *p* levels were all provided. Scale bars, 500 and 200 μm, respectively. *AP*, acute pancreatitis; *Cn*, caerulein; *CoHo*, cohoused; *Hpa-Tg*, heparanase-transgenic; *rRNA*, ribosomal RNA; *SiHo*, housed singly; *WT*, wild type

Then we conducted spontaneously microbiota-transfer study by cohousing mice, which led to the exchange of microbiota through coprophagia [27]. Mice were either housed singly (“SiHo mice”) or cohoused (“CoHo mice”) for 5 weeks before Cn injection (Fig. 4a). CoHo WT mice displayed more obvious AP phenotype compared with their SiHo WT littermate mice as presented by increased pancreas/body weight (‰), raised serum amylase, serum lipase, histologic score, serum TNF-α, and serum IL-6 levels. In contrast, CoHo WT and CoHo Hpa-Tg mice exhibited similar values in all measurements (Figure 4b–h). Consistent with the phenotype, 16S rRNA sequencing analysis showed that the microbiota composition of the CoHo WT mice was similar to that of the CoHo Hpa-Tg mice (Figure S6). Therefore, Hpa exacerbated AP in a gut microbiota-dependent manner.

### Administration of *Parabacteroides* alleviated acute pancreatitis in Hpa-Tg mice

**Fig. 5:**
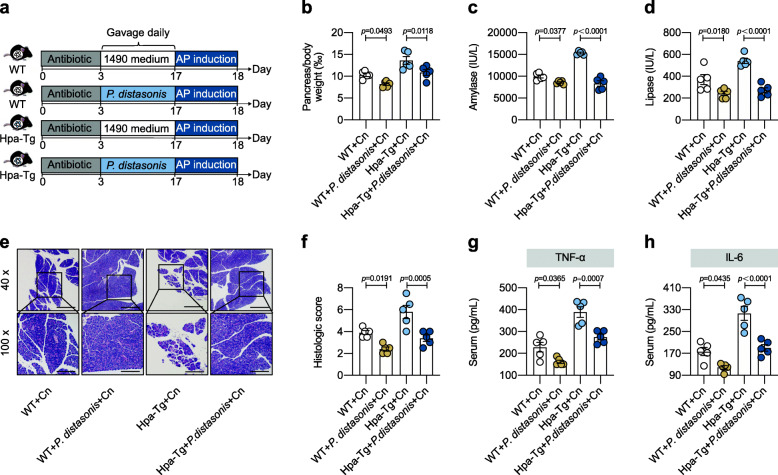
Administration of *Parabacteroides* alleviated acute pancreatitis in Hpa-Tg mice. **a** *P. distasonis* administration experimental design. WT and Hpa-Tg littermates were put on a course of intragastrically antibiotic cocktail administration for 3 days for gut microbiota depletion. Then mice were gavaged with *P. distasonis* (3 × 10^8^ CFU/ 200 μL per mouse suspended in sterile 1490 medium) daily for 2 weeks. Equivalent sterile 1490 medium was used as vehicle control. After *P. distasonis* administration, 16S rRNA sequencing analysis and AP induction were performed. **b** Pancreas (g)/body weight (g) × 1‰. **c** Serum amylase. **d** Serum lipase. **e** Representative images of pancreatic H&E staining. **f** Histologic score. **g** Serum TNF-α measured by ELISA. **h** Serum IL-6 measured by ELISA. **a–h** *n* = 5 individuals/group. Data were expressed as mean ± SEM. Differences of data were assessed by ordinary one-way ANOVA. Exact *p* levels were all provided. Scale bars, 500 and 200 μm, respectively. *AP*, acute pancreatitis; *Cn*, caerulein; *Hpa-Tg*, heparanase-transgenic; *P. distasonis*, *Parabacteroides distasonis*; *rRNA*, ribosomal RNA; *WT*, wild type

Next, we analyzed the variable importance in projection (VIP) score in an orthogonal partial least squares discrimination analysis (OPLS-DA) of the gut microbiota. We determined the commensal *Parabacteroides*, which was significantly enriched in WT mice, contributed most to distinguish the differences between WT and Hpa-Tg mice (VIP score = 2.39) (Figure S7a). *Parabacteroides* is defined as a core member of the human gut microbiota [28]. To determine if the transferred microbiota resulted in changes in *Parabacteroides*, we confirmed *Parabacteroides* abundance by all results obtained from 16S rRNA sequencing and conducted Quantitative RT-PCR (qRT-PCR) using primers specific for *Parabacteroides distasonis* (*P. distasonis* is one of the major species in *Parabacteroides* [29]). The results showed that *Parabacteroides* abundance notably changed in ABX, FMT and cohousing experiments, which was consistent with AP phenotype changes in these group (Figure S7b-d, compared to Figure S3, Figs. 3 and 4). To investigate whether *Parabacteroides* alleviated Hpa-exacerbated AP, the correlation analyses between severity indicators and *Parabacteroides* abundance were conducted. Though there were no significant correlations of *Parabacteroides* abundance with serum amylase and lipase (*R*^2^ = 0.2465 and 0.2130, *p* = 0.1041 and 0.1340, respectively), *Parabacteroides* exhibited negative correlations with pancreas/body weight (‰) (*R*^2^ = 0.6025, *p* = 0.0043), histologic score (*R*^2^ = 0.4432, *p* = 0.0209), serum TNF-α (*R*^2^ = 0.5810, *p* = 0.0055) and IL-6 (*R*^2^ = 0.4050, *p* = 0.0299) (Figure S7e-j). Then, WT and Hpa-Tg mice were gavaged with *P. distasonis* (Fig. 5a) and the colonization of *Parabacteroides* was confirmed by 16S rRNA sequencing (Figure S7k). Strikingly, compared with mice inoculated with vehicle, mice treated with *P. distasonis* displayed obviously mitigated AP, including reduced pancreas/body weight (‰), lower serum amylase, lower serum lipase, and lower histologic score (Fig. 5b–f). Levels of pro-inflammatory cytokines, including serum TNF-α and IL-6, exhibited a similar reduction in *P. distasonis*-treated mice compared with vehicle-treated mice (Fig. 5g, h). These findings suggest that the administration of *Parabacteroides* alleviated AP in Hpa-Tg mice.

### *Parabacteroides* restriction decreased acetate production in Hpa-Tg mice

**Fig. 6:**
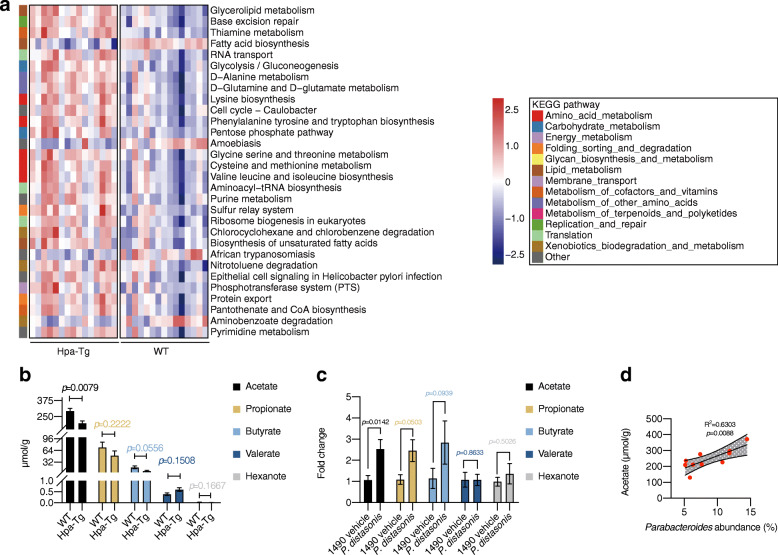
*Parabacteroides* restriction decreased acetate production in Hpa-Tg mice. **a** Annotation of microbial gene function of WT and Hpa-Tg mice on KEGG pathway analysis (Top 30). Analysis of 16S rRNA sequencing data from Fig. 2. *n* = 15 individuals/group. **b** SCFAs concentration from cecal content between healthy WT and Hpa-Tg littermates. *n* = 5 individuals/group. **c** SCFAs concentration from cecal content in 1490 vehicle group and *P. distasonis* group. *n* = 9 WT mice/group. **d** The correlation between acetate concentration and *Parabacteroides* abundance was analyzed using Spearman’s correlation. Five mice each in the WT and Hpa-Tg mice were included in the statistics. Data were expressed as mean ± SEM. Differences of data between two groups were assessed by Mann-Whitney test. Exact *p* levels were all provided. *Hpa-Tg*, heparanase-transgenic; *KEGG*, Kyoto Encyclopedia of Genes and Genomes; *P. distasonis*, *Parabacteroides distasonis*; *rRNA*, ribosomal RNA; *SCFAs*, short-chain fatty acids; *WT*, wild type

Next, we adopted the Kyoto Encyclopedia of Genes and Genomes (KEGG) pathway analysis to detect relative abundances of functional genes in gut microbiota. Sixty-nine KEGG pathways were significantly different between WT and Hpa-Tg mice (Figure S8a). Remarkably, fatty acid synthesis pathway was predominantly downregulated in Hpa-Tg mice as compared with WT mice (*p* = 1.62 × 10^-5^) (Fig. 6a). Short-chain fatty acids (SCFAs) are produced by gut microbiota fermenting dietary fiber [30] and mediate a profound anti-inflammatory effect in numerous inflammatory diseases (e.g., colitis) [31]. Therefore, we further quantified SCFA levels from WT and Hpa-Tg mice. As compared with WT mice, acetate was significantly decreased in Hpa-Tg mice, whereas, propionate, butyrate, valerate, and hexanate remained unchanged (Fig. 6b). Notably, acetate concentration changed in ABX, FMT, and cohousing experiments, which was consistent with *Parabacteroides* abundance changes in these groups (Figure S8b-d, compared to Figure S7b-d). Furthermore, the acetate level was dramatically increased after *P. distasonis* administration (Fig. 6c). *Parabacteroides* abundance is positively correlated with acetate concentration (*R*^2^ = 0.6303, *p* = 0.0088) (Fig. 6d). These results indicate that *Parabacteroides* restriction decreased acetate production in Hpa-Tg mice, and the administration of *Parabacteroides* resulted in acetate production *in vivo*.

**Fig. 7:**
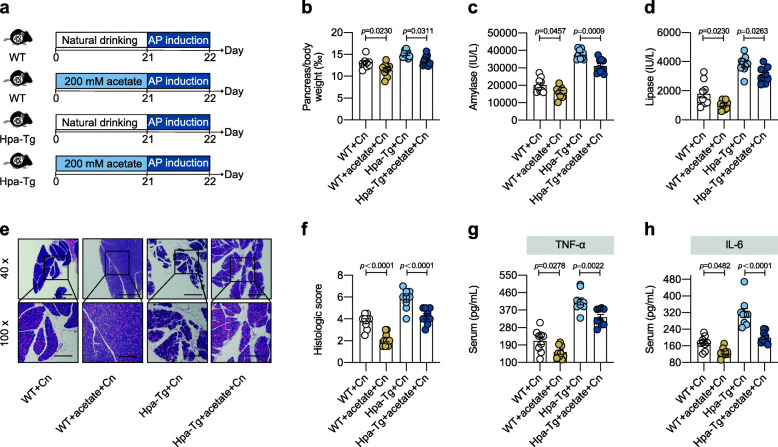
Acetate supplementation mirrored the role of *Parabacteroides* in heparanase-exacerbated acute pancreatitis. **a** Acetate supplementation experimental design. WT and Hpa-Tg littermates received normal drinking or acetate supplementation (200 mM) for 3 weeks. After acetate supplementation, AP induction were performed. **b** Pancreas (g)/body weight (g) × 1‰. **c** Serum amylase. **d** Serum lipase. **e** Representative images of pancreatic H&E staining. **f** Histologic score. **g** Serum TNF-α measured by ELISA. **h** Serum IL-6 measured by ELISA. **a–h** *n* = 5 individuals/group. Data were expressed as mean ± SEM. Differences of data were assessed by ordinary one-way ANOVA. Exact *p* levels were all provided. Scale bars, 500 and 200 μm, respectively. *AP*, acute pancreatitis; *Cn*, caerulein; *Hpa-Tg*, heparanase-transgenic; *WT*, wild type

Then, we explored whether acetate was involved in Hpa-exacerbated AP. As shown in Figure 7a, acetate was added to drinking water before AP induction. Quantification of acetate concentration was conducted to confirm the enrichment (Figure S9). Notably, acetate supplementation significantly alleviated AP (Fig. 7b–h). These results confirmed that acetate supplementation mirrored the protective role of *Parabacteroides* in Hpa-exacerbated AP.

### *Parabacteroides* produced acetate to reduce neutrophil infiltration in Hpa-exacerbated acute pancreatitis

**Fig. 8:**
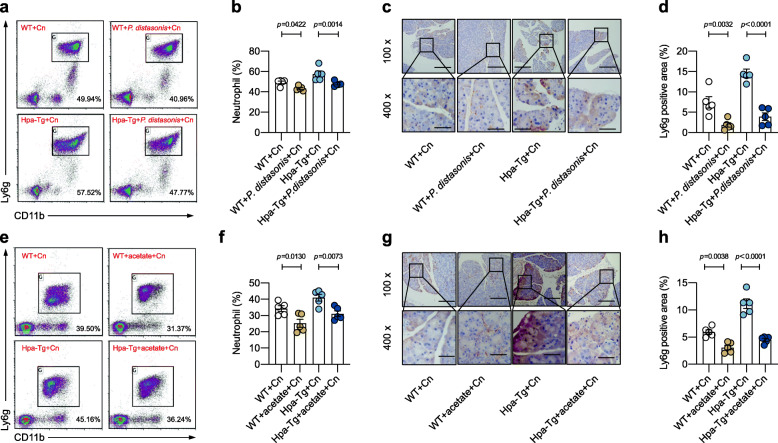
*Parabacteroides* produced acetate to reduce neutrophil infiltration in Hpa-exacerbated acute pancreatitis. **a–d** WT and Hpa-Tg littermates were treated with/without *P. distasonis* and injected with Cn to induce AP. *n* = 5 individuals/group. Representative plots of flow cytometry and bar plots of neutrophils in whole blood were shown in (**a**) and (**b**), respectively; representative plots of immunohistochemistry and bar plots of neutrophils in pancreas were shown in (**c**) and (**d**), respectively. **e** and **f** WT and Hpa-Tg littermates were treated with/without acetate and injected with Cn to induce AP. *n* = 5 individuals/group. Representative plots of flow cytometry and bar plots of neutrophils in whole blood were shown in (**e**) and (**f**), respectively. Representative plots of immunohistochemistry and bar plots of neutrophils in pancreas were shown in (**g**) and (**h**), respectively. Data were expressed as mean ± SEM. Differences of data were assessed by ordinary one-way ANOVA or Kruskal-Wallis test depending on the sample distribution. Exact *p* levels were all provided. Scale bars, 200 and 50 μm, respectively. *AP*, acute pancreatitis; *Cn*, caerulein; *Hpa-Tg*, heparanase-transgenic; *P. distasonis*, *Parabacteroides. distasonis*; *WT*, wild type

Neutrophils play a central role in the development of AP [32], and neutrophil infiltration is a rate-limiting step in the inflammatory response of AP [33]. Similarly, we found that Cn injection induced much more neutrophil infiltration in Hpa-Tg mice versus in WT mice (Figure S10a, b). Surprisingly, this difference in neutrophil infiltration disappeared after gut microbiota depletion in ABX experiment (Figure S10c, d). Moreover, increased neutrophil infiltration in Hpa-Tg mice was dependent on gut microbiota as demonstrated in FMT and cohousing experiments (Figure S10e-h). Given that *Parabacteroides* significantly alleviated Hpa-exacerbated AP, we next investigated whether *Parabacteroides* improved AP by targeting neutrophils. As we expected, *Parabacteroides* significantly decreased the number of neutrophils in blood and impaired neutrophil infiltration in the pancreas (Fig. 8a–d). We next examined the effect of acetate on neutrophils in AP. As observed in Fig. 8e–h, acetate supplementation resulted in significantly reduced neutrophils in blood and less neutrophil infiltration in the pancreas. These results suggest that *Parabacteroides* and its product acetate reduced neutrophil infiltration to alleviate pancreatic inflammation in Hpa-Tg mice.

## Discussion

**Fig. 9:**
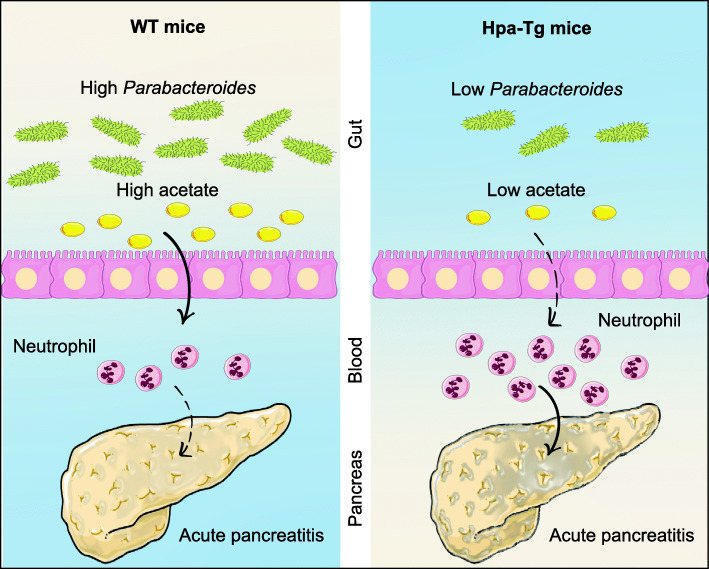
Schematic illustration for the mechanism. Mice with normal Hpa expression maintain microbiota homeostasis, and AP phenotype is not obvious. AP is exacerbated in Hpa-Tg mice, and the gut microbiota differs between Hpa-Tg mice and WT mice. The commensal *Parabacteroid*es contributes most to distinguish the differences between WT and Hpa-Tg mice. In addition, the effect of Hpa in AP is alleviated by *Parabacteroides* administrations which produces acetate to reduce neutrophil infiltration in AP. *AP*, acute pancreatitis; *Hpa-Tg*, heparanase-transgenic; *WT*, wild type

Hpa serves as a pro-inflammatory role in AP. In this study, we demonstrated a novel role for Hpa—shaping the gut microbiota. As shown in Fig. 9, we revealed that mice with normal Hpa expression maintained microbiota homeostasis, and AP was not obvious in these mice. AP was exacerbated in Hpa-Tg mice, and the gut microbiota differed between Hpa-Tg mice and WT mice. The commensal *Parabacteroid*es contributed most to distinguish the differences between WT and Hpa-Tg mice. In addition, *Parabacteroides* administration alleviated Hpa-induced AP through producing acetate to reduce neutrophil infiltration in AP. These results highlight that the gut–pancreas axis plays an important role in the development of AP, and the acetate produced by *Parabacteroides* may be beneficial for clinical treatment in AP.

Hpa is dramatically increased and exerts a pro-inflammatory role in inflammatory disease [34]. Our present study also indicated the pro-inflammatory effect of Hpa in AP, which is consistent with previous studies indicating that Hpa was involved in AP and Hpa inhibitors markedly alleviated AP in mice [11]. Studies previously demonstrated that Hpa-knockout (KO) mice compensated for the lack of Hpa by upregulating the expression levels of matrix metalloproteinase (MMP) family members, primarily MMP-2 and MMP-14. It is conceivable that MMP-2 and MMP-14, which exert some of the effects elicited by Hpa (i.e., increased mammary gland branching and enhanced angiogenic responses), compensate for its absence [35]. This compensatory mechanism prevents the induction of AP in Hpa-KO mice in the Cn-induced AP model and suggests that Hpa-Tg mice represent a more preferred model to reveal Hpa functions in AP. In this study, we focused on the mechanism by which Hpa exacerbated AP.

Accumulating evidences indicate that changes in the gut microbiota of AP patients are commonly observed [17]. The gut microbiota participates in AP progression [19, 20]. Moreover, depleting the gut microbiota by ABX alleviates AP in mice [20]. As we expected, severity indicators of AP were indistinguishable between WT and Hpa-Tg mice after ABX treatment. Transfer of microbiota from Hpa-Tg mice to WT recipient mice exacerbated AP, and Hpa-exacerbated AP was alleviated by transfer of the microbiota from WT mice. Consistent with these findings, CoHo WT and CoHo Hpa-Tg mice exhibited similar AP phenotype. Therefore, Hpa exacerbated AP in a gut microbiota-dependent manner.

Extensive studies support the existence of interactions between host genetics and the gut microbiota in diseases’ progressions, including multiple inflammatory disorders [21, 24, 36]. For example, NLRP3 shapes the gut microbiota to maintain intestinal inflammation [37] and to affect the AP severity [25]. Thus, we sought to assess whether Hpa could also shape the gut microbiota. We found that overexpression of Hpa significantly altered gut microbiota taxonomic composition. Recent studies have shown that altered *F/B* ratios are found in obesity [38], diabetes [39], etc.. Consistently, Hpa-Tg mice also exhibited a significantly increased *F/B* ratio. This finding suggests that the gut microbiota differed between Hpa-Tg mice and WT mice.

The commensal *Parabacteroid*es contributed most to distinguish the differences between WT and Hpa-Tg mice. *Parabacteroides* is reported to confer protection to multiple sclerosis [40], seizure [41], obesity, metabolic dysfunctions [42, 43], and tumors [44] as beneficial commensals. In line with that, *Parabacteroides* abundance was negatively correlated with AP phenotype. After administration of *P. distasonis*, Hpa-exacerbated AP was significantly alleviated. These findings indicate that the gut microbiota differed between Hpa-Tg mice and WT mice, and the beneficial commensal *Parabacteroides* was particularly restricted in Hpa-Tg mice.

As researches reported, bacterial translocation is strongly associated with disease progression and poor outcome in clinical pancreatitis patients [45, 46]. But there was no significant bacterial translocation to blood or pancreas in our Cn-induced model (data were not shown). Since a large number of researches have indicated that metabolites from the gut microbiota could modulate distant organs (e.g., pancreas [12], liver [47], brain [48, 49], etc.) in physiology and diseases. So we tried to explore the relationship between metabolic pathways of the gut microbiota and the Hpa-exacerbated AP. According to KEGG pathway analyses, Hpa-Tg mice displayed different levels in multiple metabolite pathways as compared with WT mice, especially fatty acid biosynthesis. SCFAs analysis showed that *Parabacteroides* restriction decreased acetate production in Hpa-Tg mice, which is consistent with studies reporting that the *Parabacteroides* is an acetate/succinate producer [29]. Increasing evidences demonstrated that SCFAs exerted a potential protective role in pancreatic functions [42] and pancreas disorders, such as diabetes [50] and pancreatitis [51–53]. Interestingly, we confirmed that acetate supplementation mirrored the protective effects of *Parabacteroides* and significantly alleviated Hpa-induced AP phenotype. This finding suggests that the beneficial role of *Parabacteroides* in AP depended on acetate production.

Neutrophils play a central role in the development of AP, and neutrophil infiltration is a rate-limiting step in the pathophysiology of AP [32]. Depletion of neutrophils or inhibition of neutrophil infiltration alleviates tissue damage in AP [33]. Consistent with these studies, we also found that the injection of Cn induced a more severe inflammatory response characterized by neutrophil infiltration in Hpa-Tg mice as compared with WT mice. Recent studies have indicated that antibiotic exposure decreased numbers of circulating and bone marrow neutrophils, and the gut microbiota regulates neutrophil homeostasis [54, 55]. Consistent with these studies, we also revealed that difference in neutrophil infiltration between WT and Hpa-Tg mice disappeared after gut microbiota depletion. Furthermore, increased neutrophil infiltration in Hpa-Tg mice was gut microbiota transferable in FMT and cohousing experiments. These data indicated that Hpa increased neutrophil infiltration to exacerbate AP in a gut microbiota-dependent manner.

Recent studies have revealed that bacterial metabolites, such as SCFAs, are not always restricted to the intestine, but may enter the circulation and affect cells located in peripheral tissues [56]. Thus, SCFAs potentially regulate disorders of distant organs (such as the pancreas). In particular, microbiota-derived SCFAs play an essential role in anti-inflammatory responses through innate and adaptive immune cells locally and systemically [57]. Moreover, acetate is the most abundant SCFAs in peripheral circulation [30]. Previous studies have demonstrated that acetate exerts anti-inflammatory effects through regulating multiple immune cells, especially negatively regulating neutrophils. For example, acetate alleviated inflammation in gout arthritis by inducing caspase-dependent neutrophil apoptosis to reduce neutrophil infiltration [58]. During respiratory syncytial viral (RSV) infection, acetate reduces neutrophil numbers to mitigate inflammation [59]. Similarly, we demonstrated in Hpa-Tg mice that acetate supplementation or administration of the acetate producing commensal *Parabacteroides* significantly decreased the number of neutrophils in blood and impaired neutrophil infiltration in the pancreas. Previous study reported that acetate could bind and activate the G-protein-coupled receptor GPR43 on neutrophils to induce its apoptosis [60]. Further studies are needed to explore the role of Hpa and *Parabacteroides* in acetate-GPR43-mediated neutrophil apoptosis in AP.

## Conclusions

In conclusion, our investigation attempts to provide comprehensive information on the interaction between gut microbiota and Hpa in AP. We found that Hpa shapes the composition and function of the gut microbiota. The gut–pancreas axis plays an important role in Hpa-exacerbated AP. Furthermore, the administration of *Parabacteroides* or acetate supplementation provides a novel therapeutic strategy to treat AP.

## Methods

### Animals

C57 BL/6 wild-type (WT) mice were obtained from Chongqing Tengxin Biotechnology Co. Ltd. (Chongqing, China). Heparanase transgenic (Hpa-Tg) mice overexpressing human Hpa in a C57BL/6 genetic background were previously generated [61]. Across all experimental conditions, WT and Hpa-Tg mice were originally generated from the same breeders and raised in the same facilities (at Third Military Medical University) for more than nine generations. Age- and sex-matched WT and Hpa-Tg littermates were used. Throughout the experimental periods, all mice were cared for in a specific pathogen-free (SPF) environment with controlled conditions, including a 12-h light/dark cycle at 20–22 °C and 45 ± 5% humidity.

### Caerulein (Cn)-induced acute pancreatitis (AP)

Age- and sex-matched WT and Hpa-Tg littermates originating from the same breeders were used for AP induction. Briefly, mice fasted for 12 h and were then injected with either Cn (intraperitoneally, 50 μg/kg body weight, 8 times at 1-h intervals) (Tocris Bioscience, Cat. No. 6264, Bristol, UK) or sterile water. Mice were sacrificed 1 h after the last injection. Pancreatic tissue was isolated and weighted. Blood, intestinal tissue, cecal content, and feces were collected for further measurements.

### Serum amylase and lipase

After standing for 4 h, blood was centrifuged (1500 rpm at room temperature) for 10 min. Serum was collected as supernatant. Amylase and lipase levels were detected using the fully automatic biochemistry analyzer in the laboratory department of the Third Military Medical University Second Affiliated Hospital.

### Histopathology

Pancreatic tissue was fixed in 10% neutral-buffered formalin, paraffin embedded and processed for histological analysis. Five-micron sections were stained with H&E and semiquantitatively scored using Schmidt’s criteria (Table S1) by two board-certified veterinary pathologists in a blinded manner. The final score is expressed as the average of these two values.

### Quantification of cytokine levels

Serum was used to detect cytokines TNF-α (Beyotime, Cat. No. PT512) and IL-6 (Beyotime, Cat. No. PI326) levels by ELISA analysis according to the manufacturer’s instructions.

### RNA extraction and quantitative RT-PCR (qRT-PCR) (SYBR Green)

Intestinal tissue was preserved in liquid nitrogen. Total RNA was extracted with Trizol reagent (Takara, Cat. No. 9109, Kusatsu, Japan). cDNA was synthesized with PrimeScript RT reagent Kit (Takara, Cat. No. RR047A, Kusatsu, Japan). qRT-PCR was performed on cDNA with TB Green Premix Ex Taq II kit (Takara, Cat. No. RR820A, Kusatsu, Japan) in a 20-μL volume on an ABI 7900 HT Fast Real-Time cycler (Applied Biosystems, Foster city, USA) according to the manufacturer’s instruction using the housekeeping gene β-actin (for mouse) as a control. The specific primers are listed in Table S2.

### Fecal genomic DNA extraction and 16S ribosomal RNA (rRNA) sequencing

Fecal genomic DNA was extracted from approximately 100 mg of frozen fecal samples from mice using the TIANamp Stool DNA Kit (TIANGEN, Cat. No. DP328, Beijing, China) according to the manufacturer’s instructions. The concentration and purity of the extracted bacterial DNA were measured using a NanoDrop 2000C spectrophotometer (Thermo Fisher Scientific, Waltham, USA). The 16S rRNA gene V3-V4 region-specific primers are 341F: CCTAYGGGRBGCASCAG, 806R: GGACTACNNGGGTATCTAAT. The PCR products were purified using the GeneJET Gel Extraction Kit (Thermo Fisher Scientific, Cat. No. K0691, Waltham, USA). The libraries were sequenced using the Illumina NovaSeq 6000 platform by Novogene (Tianjin, China). The Quantitative Insights into Microbial Ecology 2 (QIIME2, version 2020. 2 [62]) platform within a conda environment was used to process the sequencing data. Briefly, the V3-V4 primers of paired-end fastq format sequence files were trimmed by using *cutadapt* 3.1 [63] with Python 3.6.9. Then, trimmed fastq files were imported into QIIME2 by using “qiime tools import” command. By using “qiime dada2 denoise-paired” command, reads were denoised into amplicon sequence variants (ASVs), and represent sequence of each ASV and feature table were generated. In addition, DADA2 [64] contains internal chimera checking methods and abundance filtering, so additional filtering should not be necessary. After the DADA2 denoising step completed, the “qiime feature-table summarize” command was used to generate a feature table summary report listing how many sequences are associated with each sample and with each feature. Samples with less than 8000 sampling depth (non-chimeric sequences) were excluded from further analysis. “qiime feature-table rarefy” command was used to rarefy all samples into same depth, and a rarefied feature table was generated. All rarefaction curves are provided in Figure S11. After rarefaction, alpha diversity and beta diversity were calculated by “qiime diversity alpha” and “qiime diversity beta” command from the rarefied feature table, respectively. After computing diversity metrics, the principal coordinate analysis (PCoA analysis) results were calculated by “qiime diversity pcoa” command and visualized by “qiime emperor plot” command. Analysis of similarities (ANOSIM) test was performed to calculate significance of dissimilarity. A pre-trained Naive Bayes classifier (silva-132-99-nb-classifier.qza, trained on SILVA [65] 132 99% full-length sequences) and the “qiime feature-classifier classify-sklearn” command were used to explore the taxonomic composition of the samples. The predominance of bacterial communities between groups was analyzed using the linear discriminant analysis (LDA) effect size (LEfSe) (http://huttenhower.sph.harvard.edu/lefse) [66] (LDA score (log10) = 3.5 as cutoff value). Orthogonal partial least squares discrimination analysis (OPLS-DA) and the variable importance in projection (VIP) score (1.5 as cutoff value) evaluations were performed using R package *ropls* [67]. Phylogenetic Investigation of Communities by Reconstruction of Unobserved States (PICRUSt2) [68] was used for predicting functional abundances including Kyoto Encyclopedia of Genes and Genomes (KEGG) orthologs and KEGG pathways based on marker gene sequences (16S gene).

### Antibiotic cocktail (ABX) experiment

Age- and sex-matched WT and Hpa-Tg littermates originating from the same breeders received a four-antibiotic cocktail (neomycin sulfate 200 mg/kg, metronidazole 200 mg/kg, ampicillin 200 mg/kg, and vancomycin 100 mg/kg) intragastrically once daily for 5 days to deplete the gut microbiota. All the antibiotics used here were purchased from Sangon Biotech (Cat. Nos. A610366, A600633, A610028, and A600983; Shanghai, China).

### Fecal microbiota transplantation (FMT) experiment

For the fecal microbiota transplantation experiment, age- and sex-matched WT and Hpa-Tg littermates originating from the same breeders were pretreated with a four-antibiotic cocktail (as previously described) to clear gut microbiota followed by oral gavage daily for 8 days with a PBS suspension of feces (200 μL per mouse) derived from WT or Hpa-Tg donor mice. The mice were injected with Cn 1 day after the final gavage.

### Cohousing experiment

For cohousing experiments, age- and sex-matched WT and Hpa-Tg littermates originating from the same breeders were divided to be either housed singly (SiHo WT) (Cage No. 1) or cohoused with age- and sex-matched mice (CoHo WT and CoHo Hpa-Tg mice at 1:1 ratio) (Cage No. 2) for 5 weeks. Mice were injected with Cn 1 day after the cohousing period was complete.

### Microbial strain

*Parabacteroides distasonis* (*P. distasonis*) was purchased from American Type Culture Collection (ATCC, Cat. No. 8503, Rockville, USA) and cultured in 1490 medium under anaerobic conditions using an anaerobic chamber (GeneScience, E500, USA) according to the manufacturer’s instructions. To detect the role of *P. distasonis* in AP, age- and sex-matched WT and Hpa-Tg littermates originating from the same breeders received a four-antibiotic cocktail (as previously described) once a day for 3 days to deplete the gut microbiota and then treated by oral gavage with *P. distasonis* (3 × 10^8^ CFU/200 μL per mouse suspended in sterile 1490 medium) daily for 2 weeks. Equivalent sterile 1490 medium was used as vehicle control. The colonization of *P. distasonis* was confirmed by qRT-PCR. The mice were administered a Cn injection 1 day after the final gavage.

### DNA extraction and qRT-PCR (Taqman)

Intestinal tissue DNA was extracted with TIANamp Micro DNA Kit (TIANGEN, Cat. No. DP316, Beijing, China). qRT-PCR was performed on DNA with Premix Ex Taq^TM^ (Probe qPCR) (Takara, Cat. No. RR390A, Kusatsu, Japan) in a 20-μL volume on an ABI 7900 HT Fast Real-Time cycler (Applied Biosystems, Foster city, USA) according to the manufacturer’s instruction using the housekeeping gene GAPDH (for mouse) as a control. The specific primers of GAPDH and *P. distasonis* are from previous studies [69, 70] and listed in Table S3.

### Short-chain fatty acid (SCFA) quantification analysis

Cecal content (20 mg per mouse) (excluding intestinal tissue) were supplemented with grinding beads and 10% isobutanol aqueous solution and centrifuged. Then, isobutanol, pyridine, and ultrapure water were added to the supernatant. Isobutyl chloroformate was added for derivatization. The extracted samples were detected using a 7890B-5977, GC-MS system (Agilent Technologies, Santa Clara, USA). SCFA standards were mixtures of acetate, propionate, butyrate, valerate, and hexanoate. All the standards were purchased from Merck (Darmstadt, Germany).

### Sodium acetate supplementation

Briefly, 200 mM sodium acetate (Sigma-Aldrich, Cat. No. S5636, St. Louis, USA) was dissolved in drinking water, sterilized, freshly prepared, and changed every 3 days. Age- and sex-matched WT and Hpa-Tg littermates originating from the same breeders received normal drinking or acetate supplementation for 3 weeks before AP induction.

### Neutrophil detection by flow cytometry

Blood was anticoagulated by 15 g/L EDTA-Na_2_ and then centrifuged at 1500 rpm for 5 min to remove plasma. Cell populations were resuspended in precooling PBS and then stained using APC anti-mouse Ly-6 g antibody (BioLegend, Cat. No. 127614, dilution 1:100, San Diego, USA) and Brilliant Violet 421 anti-mouse/human CD11b antibody (BioLegend, Cat. No. 121035, dilution 1:100, San Diego, USA) for 30 min at 4 °C. Red blood cells were removed with Red Blood Cell Lysis Buffer (Solarbio, Cat. No. R1010, Beijing, China). Flow cytometry was performed on the Beckman Coulter platform (CA, USA). The neutrophil proportion was analyzed using Kaluza software (Version 2.1).

### Immunohistochemistry analysis

Neutrophil quantitation was performed after immunohistochemistry with an Anti-Ly6 g monoclonal antibody (Abcam, Cat. No. 25377, Cambridge, UK). Paraformaldehyde-fixed and paraffin-embedded samples were cut into 5-μm sections. Antigen retrieval was achieved by heating. Samples were incubated with primary antibody (1/100 in 1% BSA in PBS) for 12 h at 4 °C. Protein expression was quantified using image J software (National Institutes of Health; http://www.imagej.softonic.de).

### Statistical analysis

Statistical analysis was performed with GraphPad Prism 8.0 software (GraphPad Software Inc., San Diego, USA). Data were expressed as the means ± SEM and considered significant at *p* < 0.05. Exact *p* levels in all figures were provided. The normality of distribution was determined using Shapiro-Wilk test or Kolmogorov-Smirnov test. Homogeneity of variance was tested by F test (for two groups) or Brown-Forsythe test (for more than two groups). Significance between two groups was determined by unpaired, two-tailed *t* test or Mann-Whitney test depending on the sample distribution. Significance between multiple groups was determined through ordinary one-way ANOVA or Kruskal-Wallis test depending on the sample distribution, and a post hoc Tukey test was used to do multiple comparisons test. Correlation analyses were conducted using Spearman’s correlation.

## Supplementary Information

**Additional file 1: Supplemental Figure 1.** Confirmation of mouse and human Hpa encoding genes expressions across all experimental conditions. (a) Genotype identification of Hpa- Tg mice. Semiquantitative PCR amplification of DNA extracted from WT (n=1) and Hpa-Tg mice (n=10). Human Hpa specific primer was used to detect transgene vector sequences in the mouse genome. Total DNA was assessed by specific primer against a genomic sequence of the ribosomal protein L-19. The expression levels of mouse Hpa (b) and human Hpa (c) in WT+SW/WT+Cn/Hpa-Tg+SW/Hpa-Tg+Cn group were confirmed. The expression levels of mouse vs human Hpa in in Hpa-Tg SW/HpaTg+Cn groups were confirmed (d). (e) Before 16S rRNA sequencing of WT and HpaTg mice, the expression levels of mouse Hpa and human Hpa were confirmed. The expression levels of mouse or human Hpa, the expression levels of mouse vs human Hpa were also confirmed in ABX (f), FMT (g-i), cohousing (j), *Parabacteroides a*dministration (k-m) and acetate supplementation (n-p) experiments. Data were expressed as mean ± SEM. Significance between two groups was determined by unpaired, two-tailed t test or Mann-Whitney test depending on the sample distribution. Significance between multiple groups was determined through ordinary one-way ANOVA or Kruskal-Wallis test depending on the sample distribution. Exact *p* levels were all provided. ABX, antibiotic cocktail; Cn, caerulein; CoHo, cohoused; FMT, fecal microbiota transplantation; Hpa-Tg, heparanase-transgenic; *P. distasonis*, *Parabacteroides distasonis*; SiHo, housed singly; SW, sterile water; rRNA, ribosomal RNA; WT, wild-type. **Supplemental Figure 2.** Alpha-diversity, beta-diversity and bar plots of the phylum/class/order/family/genus taxonomic levels in WT and Hpa-Tg mice. (a) Alpha-diversity (Based on ACE, Observed features, Fisher alpha, Shannon and Faith pd). (b) PCoA of beta-diversity using Jaccard, Generalized Unifrac and Weighted Unifrac metric distance. (c) Quantification of dissimilarity values based on (b), presented as dissimilarity values (first (box bottom), third (box top) quartiles, the median (line inside box) and 2.5 interquartile range (line ends)). (d) Bar plots of the phylum taxonomic levels in WT and Hpa-Tg mice. Relative abundance is plotted for each group. The relative abundances of *Firmicutes* and *Bacteroidetes* were shown. (e) Bar plots of the class taxonomic levels in WT and Hpa-Tg mice. Relative abundance is plotted for each group. (f) Bar plots of the order taxonomic levels in WT and Hpa-Tg mice. Relative abundance is plotted for each group. Bacterial genera with relative abundance greater than 0.01% were analyzed. (g) Bar plots of the family taxonomic levels in WT and Hpa-Tg mice. Relative abundance is plotted for each group. Bacterial genera with relative abundance greater than 0.1% were analyzed. (h) Bar plots of the genus taxonomic levels in WT and Hpa-Tg mice. Relative abundance is plotted for each group. Bacterial genera with relative abundance greater than 1% were analyzed. Data were expressed as mean ± SEM. n=15 individuals/group. Significance between two groups was determined by unpaired, two-tailed t test or Mann-Whitney test depending on the sample distribution. For (b-c), differences of data were assessed by ANOSIM test. Exact *p* levels were all provided. ANOSIM, analysis of similarities Hpa-Tg, heparanase-transgenic; WT, wild-type. **Supplemental Figure 3.** Exacerbated acute pancreatitis in Hpa-Tg mice depended on gut microbiota. (a) ABX experimental design, WT and Hpa-Tg littermates were put on a course of intragastrically antibiotic cocktail administration for 5 days for gut microbiota depletion After that, 16S rRNA sequencing analysis and AP induction were performed. (b) Pancreas (g)/body (g) weight x 1‰. (c) Serum amylase. (d) Serum lipase. (e) Representative images of pancreatic H&E staining. (f) Histologic score. (g) Serum TNF-α measured by ELISA. (h) Serum IL-6 measured by ELISA. (a-h) n=5 individuals/group. Data were expressed as mean ± SEM. Differences of data were assessed by assessed by unpaired, two-tailed t test or Mann-Whitney test depending on the sample distribution. Exact *p* levels were all provided. Scale bars, 500 and 200 μm, respectively. ABX, antibiotic cocktail; AP, acute pancreatitis; Cn, caerulein; Hpa-Tg, heparanase-transgenic; rRNA, ribosomal RNA; WT, wild-type. **Supplemental Figure 4.** Beta-diversity among ABX experimental groups. 16S rRNA sequencing analysis in fecal bacterial DNA from ABX WT mice (n=5) and Hpa Tg mice (n=3) was performed. PCoA of beta-diversity using Bray-Curtis (a), Jaccard (b), Generalized Unifrac (c) and Weighted Unifrac (d) metric distance. Differences of data were assessed by ANOSIM test. Exact *p* levels were all provided. ABX, antibiotic cocktail; ANOSIM, analysis of similarities; Hpa-Tg, heparanase-transgenic; PCoA, principal coordinate analysis; rRNA, ribosomal RNA; WT, wild-type. **Supplemental Figure 5.** Beta-diversity among FMT experimental groups. 16S rRNA sequencing analysis in fecal bacterial DNA from FMT groups was performed. n=7 individuals/group. PCoA of beta-diversity using Bray-Curtis (a), Jaccard (b), Generalized Unifrac (c) and Weighted Unifrac (d) metric distance. Differences of data were assessed by ANOSIM test. Exact *p* levels were all provided. ANOSIM, analysis of similarities; Hpa-Tg, heparanase-transgenic; PCoA, principal coordinate analysis FMT, fecal microbiota transplantation; rRNA, ribosomal RNA; WT, wild-type. **Supplemental Figure 6.** Beta-diversity among CoHo WT and CoHo Hpa-Tg groups. 16S rRNA sequencing analysis in fecal bacterial DNA from CoHo WT and CoHo Hpa-Tg groups was performed. n=7 individuals/group. PCoA of beta-diversity using Bray-Curtis **(a)**, Jaccard **(b)**, Generalized Unifrac **(c)** and Weighted Unifrac **(d)** metric distance. Differences of data were assessed by ANOSIM test. Exact *p* levels were all provided. ANOSIM, analysis of similarities; CoHo, cohoused; Hpa-Tg, heparanase-transgenic; PCoA, principal coordinate analysis; rRNA, ribosomal RNA; WT, wild-type. **Supplemental Figure 7.** *Parabacteroides* was the most important biomarker to distinguish WT and Hpa-Tg mice. **(a)** VIP score of OPLS-DA. VIP score (calculated based on 16S rRNA sequencing data of Fig. 2) was used to rank the ability of different taxa to discriminate between WT and Hpa-Tg mice. A taxon with VIP score >1.5 was considered important in the discrimination. n=15 individuals/group. (b) In ABX experiment, *Parabacteroides* abundance was confirmed by 16S rRNA sequencing (n=5/3 in ABX WT/ABX Hpa-Tg group, respectively) and qRT-PCR (using specific primers of *P. distasonis*, n=5 individuals/group). (c) In FMT experiment, *Parabacteroides* abundance was confirmed by 16S rRNA sequencing and qRT-PCR (using specific primers of *P. distasonis*). n=7 individuals/group. (d) In Cohousing experiment, *Parabacteroides* abundance was confirmed by 16S rRNA sequencing and qRT-PCR (using specific primers of *P. distasonis*). n=7 individuals/group. (e-j) The correlations between severity indicators of AP and *Parabacteroides* abundance were analyzed using Spearman’s correlations. 6 mice each in WT and Hpa-Tg mice were included in the statistics. (k) In *Parabacteroides* administration experiment, *Parabacteroides* abundance was confirmed by 16S rRNA sequencing. n=5 individuals/group. Data were expressed as mean ± SEM. Differences of data in two groups were assessed by assessed by unpaired, two-tailed t test or Mann-Whitney test depending on the sample distribution. Differences of data in more than two groups were assessed by ordinary one-way ANOVA or Kruskal-Wallis test depending on the sample distribution. Exact *p* levels were all provided. ABX, antibiotic cocktail; AP, acute pancreatitis; FMT, fecal microbiota transplantation; Hpa-Tg, heparanase-transgenic; OPLS-DA, orthogonal partial least squares discrimination analysis; *P. distasonis*, *Parabacteroides. distasonis*; qRT-PCR, Quantitative RT-PCR; rRNA, ribosomal RNA; VIP, valuable influence on projection; WT, wild-type. **Supplemental Figure 8.** Acetate concentration in ABX, FMT and cohousing experiments. (a) The whole annotation of microbial gene function of WT and Hpa-Tg mice on KEGG pathway analysis. Analysis of 16S rRNA sequencing data from Figure 2. n = 15 individuals/group. (b) Relative fold change of acetate concentration from cecal content in ABX experiment. n=5 individuals/group. (c) Relative fold change of acetate concentration from cecal content in FMT experiment. n=7 individuals/group. (d) Relative fold change of acetate concentration from cecal content in CoHo WT and CoHo Hpa-Tg mice. n=7 individuals/group. Data were expressed as mean ± SEM. Differences of data in two groups were assessed by assessed by Mann-Whitney test. Differences of data in FMT experiment was assessed by ordinary one-way ANOVA. Exact *p* levels were all provided. ABX, antibiotic cocktail; CoHo, cohoused; FMT, fecal microbiota transplantation; Hpa-Tg, heparanase-transgenic; rRNA, ribosomal RNA; KEGG, kyoto encyclopedia of genes and genomes; WT, wild-type. **Supplemental Figure 9.** Confirmation of acetate enrichment in acetate supplementation experiment. Relative fold change of acetate concentration from cecal content in acetate supplementation experiment. n=9 individuals/group. Data were expressed as mean ± SEM. Differences of data were assessed by ordinary one-way ANOVA. Exact *p* levels were all provided. WT, wild-type; Hpa-Tg, heparanase transgenic. **Supplemental Figure 10.** Neutrophil infiltration depended on gut microbiota in heparanase-exacerbated acute pancreatitis. WT and Hpa-Tg mice were treated with/without Cn. Representative plots of immunohistochemistry and bar plots of neutrophils in pancreas were shown in (a) and (b), respectively. Representative plots of immunohistochemistry and bar plots of neutrophils in pancreas of ABX experiment were shown in (c) and (d), respectively. Representative plots of immunohistochemistry and bar plots of neutrophils in pancreas of FMT experiment were shown in (e) and (f), respectively. Representative plots of immunohistochemistry and bar plots of neutrophils in pancreas of cohousing experiment were shown in (g) and (h), respectively. Differences of data in two groups were assessed by unpaired, two-tailed t test. Differences of data in more than two groups were assessed by ordinary one-way ANOVA. Exact *p* levels were all provided. Scale bars, 200 and 50 μm, respectively. ABX, antibiotic cocktail; FMT, fecal microbiota transplantation; Hpa-Tg, heparanase- X transgenic; Cn, caerulein; CoHo, cohoused. SiHo, housed singly; SW, sterile water; WT, wild-type. **Supplemental Figure 11.** Alpha rarefaction curves of all 16S rRNA sequencing analyses. Alpha rarefaction curves (Faith pd) in WT and Hpa-Tg groups (a), ABX experiment (b), CoHo WT and CoHo Hpa-Tg groups (c), FMT experiment (d) and the administration of *Parabacteroides* experiment (e). ABX, antibiotic cocktail; CoHo, cohoused; FMT, fecal microbiota transplantation; Hpa-Tg, heparanase-transgenic; *P. distasonis*, *Parabacteroides distasonis*; WT, wild-type. **Supplemental Table 1.** Schmidt's score system of pancreatic histopathology. **Supplemental Table 2.** Primers for qRT-PCR detection (SYBR Green). **Supplemental Table 3.** Primers for qRT-PCR detection (Taqman).

## Data Availability

Raw 16S rRNA sequencing data have been deposited in The European Nucleotide Archive (http://www.ebi.ac.uk/ena) with Study No. PRJEB42924. The other data are available from the corresponding author upon reasonable request.

## Abbreviations

ABX: Antibiotic cocktail
ANOSIM: Analysis of similarities
AP: Acute pancreatitis
ASVs: Amplicon sequence variants
Cn: Caerulein
CoHo: Cohoused
CRAMP: Cathelicidin-related antimicrobial peptide
DEFB1: β-defensin 1
*F/B*: *Firmicutes*/*Bacteroidetes*
FMT: Fecal microbiota transplantation
GEO: Gene Expression Omnibus database
Hpa-Tg: Heparanase-transgenic
HS: Heparan sulfate
KEGG: Kyoto Encyclopedia of Genes and Genomes
KO: Knockout
LDA: Linear discriminant analysis
LEfSe: Linear discriminant analysis effect size
MMP: Matrix metalloproteinase
OPLS-DA: Orthogonal partial least squares discrimination analysis
PCoA: Principal coordinate analysis
PICRUSt2: Phylogenetic investigation of communities by reconstruction of unobserved states
QIIME2: Quantitative insights into microbial ecology 2
qRT-PCR: Quantitative RT-PCR
*P. distasonis*: *Parabacteroides distasonis*
rRNA: Ribosomal RNA
RSV: Respiratory syncytial viral
SCFAs: Short-chain fatty acids
SiHo: Housed singly
SPF: Specific pathogen-free
SW: Sterile water
VIP: Variable importance in projection
WT: Wild type
