# Supplementary material for: Parabacteroides produces acetate to alleviate heparanase-exacerbated acute pancreatitis through reducing neutrophil infiltration
Source: Microbiome. 2021 May 20;9:115. doi: 10.1186/s40168-021-01065-2 (PMC8138927; doi:10.1186/s40168-021-01065-2)
Supplement: Supplementary file 2 — Additional file 1: Supplemental Figure 1. Confirmation of mouse and human Hpa encoding genes expressions across all experimental conditions. (a) Genotype identification of Hpa- Tg mice. Semiquantitative PCR amplification of DNA extracted from WT (n=1) and Hpa-Tg mice (n=10). Human Hpa specific primer was used to detect transgene vector sequences in the mouse genome. Total DNA was assessed by specific primer against a genomic sequence of the ribosomal protein L-19. The expression levels of mouse Hpa (b) and human Hpa (c) in WT+SW/WT+Cn/Hpa-Tg+SW/Hpa-Tg+Cn group were confirmed. The expression levels of mouse vs human Hpa in in Hpa-Tg SW/HpaTg+Cn groups were confirmed (d). (e) Before 16S rRNA sequencing of WT and HpaTg mice, the expression levels of mouse Hpa and human Hpa were confirmed. The expression levels of mouse or human Hpa, the expression levels of mouse vs human Hpa were also confirmed in ABX (f), FMT (g-i), cohousing (j), Parabacteroides administration (k-m) and acetate supplementation (n-p) experiments. Data were expressed as mean ± SEM. Significance between two groups was determined by unpaired, two-tailed t test or Mann-Whitney test depending on the sample distribution. Significance between multiple groups was determined through ordinary one-way ANOVA or Kruskal-Wallis test depending on the sample distribution. Exact p levels were all provided. ABX, antibiotic cocktail; Cn, caerulein; CoHo, cohoused; FMT, fecal microbiota transplantation; Hpa-Tg, heparanase-transgenic; P. distasonis, Parabacteroides distasonis; SiHo, housed singly; SW, sterile water; rRNA, ribosomal RNA; WT, wild-type. Supplemental Figure 2. Alpha-diversity, beta-diversity and bar plots of the phylum/class/order/family/genus taxonomic levels in WT and Hpa-Tg mice. (a) Alpha-diversity (Based on ACE, Observed features, Fisher alpha, Shannon and Faith pd). (b) PCoA of beta-diversity using Jaccard, Generalized Unifrac and Weighted Unifrac metric distance. (c) Quantification of dissimilarity v [file 40168_2021_1065_MOESM2_ESM.pdf]

Supplementary materials

Parabacteroides produces acetate to alleviate heparanase-exacerbated acute pancreatitis through reducing neutrophil infiltration

Yuanyuan Lei[1†], Li Tang[1†], Shuang Liu[1], Shiping Hu[1,2], Lingyi Wu[1,3], Yaojiang Liu[1], Min Yang[1], Shengjie Huang[1,4], Xuefeng Tang[5], Tao Tang[6], Xiaoyan Zhao[1], Israel Vlodavsky[7], Shuo Zeng[1], Bo Tang[1]*, Shiming Yang[1]*

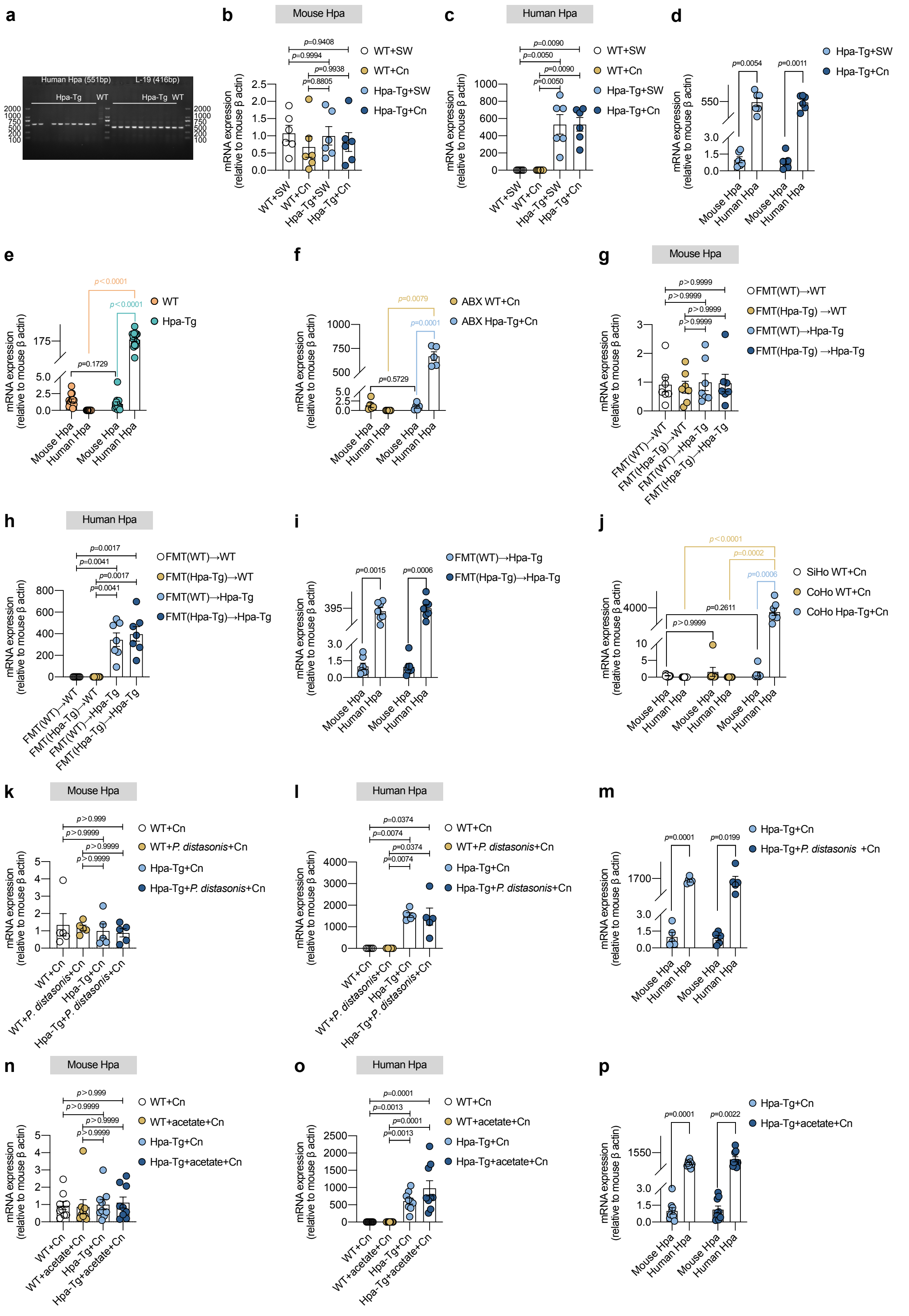

Supplemental Figure 1. Confirmation of mouse and human Hpa encoding genes expressions across all experimental conditions. (a) Genotype identification of Hpa-

Tg mice. Semiquantitative PCR amplification of DNA extracted from WT (n=1) and Hpa-Tg mice (n=10). Human Hpa specific primer was used to detect transgene vector sequences in the mouse genome. Total DNA was assessed by specific primer against a genomic sequence of the ribosomal protein L-19. The expression levels of mouse Hpa **(b)** and human Hpa **(c)** in WT+SW/WT+Cn/Hpa-Tg+SW/Hpa-Tg+Cn group were confirmed. The expression levels of mouse vs human Hpa in in Hpa-Tg SW/Hpa-Tg+Cn groups were confirmed **(d)**. **(e)** Before 16S rRNA sequencing of WT and Hpa-Tg mice, the expression levels of mouse Hpa and human Hpa were confirmed. The expression levels of mouse or human Hpa, the expression levels of mouse vs human Hpa were also confirmed in ABX **(f)**, FMT **(g-i)**, cohousing **(j)**, *Parabacteroides* administration **(k-m)** and acetate supplementation **(n-p)** experiments. Data were expressed as mean ± SEM. Significance between two groups was determined by unpaired, two-tailed t test or Mann-Whitney test depending on the sample distribution. Significance between multiple groups was determined through ordinary one-way ANOVA or Kruskal-Wallis test depending on the sample distribution. Exact *p* levels were all provided. ABX, antibiotic cocktail; Cn, caerulein; CoHo, cohoused; FMT, fecal microbiota transplantation; Hpa-Tg, heparanase-transgenic; *P. distasonis*, *Parabacteroides distasonis*; SiHo, housed singly; SW, sterile water; rRNA, ribosomal RNA; WT, wild-type.

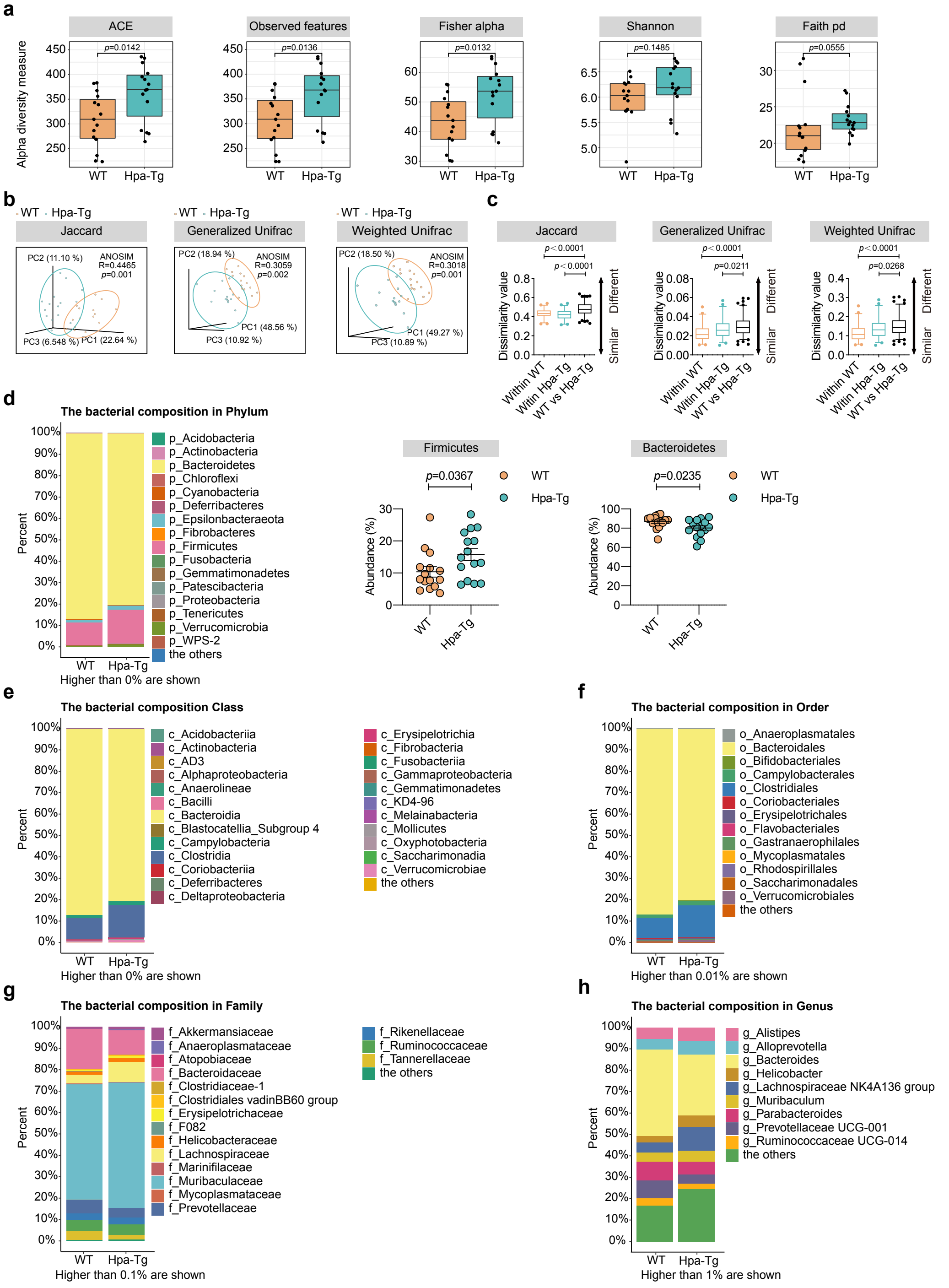

Supplemental Figure 2. Alpha-diversity, beta-diversity and bar plots of the phylum/class/order/family/genus taxonomic levels in WT and Hpa-Tg mice. (a) Alpha-diversity (Based on ACE, Observed features, Fisher alpha, Shannon and Faith pd). **(b)** PCoA of beta-diversity using Jaccard, Generalized Unifrac and Weighted

Unifrac metric distance. **(c)** Quantification of dissimilarity values based on (b), presented as dissimilarity values (first (box bottom), third (box top) quartiles, the median (line inside box) and 2.5 interquartile range (line ends)). **(d)** Bar plots of the phylum taxonomic levels in WT and Hpa-Tg mice. Relative abundance is plotted for each group. The relative abundances of *Firmicutes* and *Bacteroidetes* were shown. **(e)** Bar plots of the class taxonomic levels in WT and Hpa-Tg mice. Relative abundance is plotted for each group. **(f)** Bar plots of the order taxonomic levels in WT and Hpa-Tg mice. Relative abundance is plotted for each group. Bacterial genera with relative abundance greater than 0.01% were analyzed. **(g)** Bar plots of the family taxonomic levels in WT and Hpa-Tg mice. Relative abundance is plotted for each group. Bacterial genera with relative abundance greater than 0.1% were analyzed. **(h)** Bar plots of the genus taxonomic levels in WT and Hpa-Tg mice. Relative abundance is plotted for each group. Bacterial genera with relative abundance greater than 1% were analyzed. Data were expressed as mean ± SEM. n=15 individuals/group. Significance between two groups was determined by unpaired, two-tailed t test or Mann-Whitney test depending on the sample distribution. For **(b-c)**, differences of data were assessed by ANOSIM test. Exact *p* levels were all provided. ANOSIM, analysis of similarities Hpa-Tg, heparanase-transgenic; WT, wild-type.

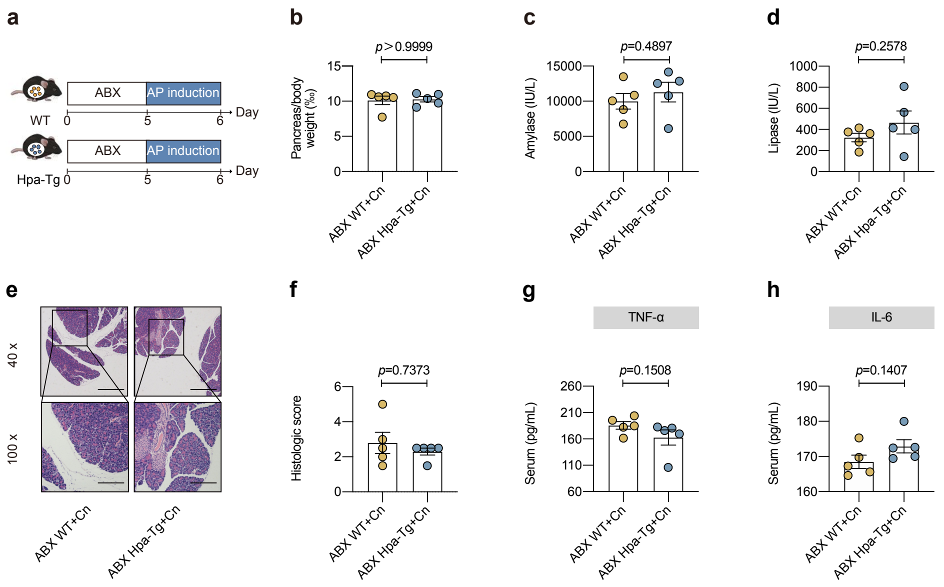

Supplemental Figure 3. Exacerbated acute pancreatitis in Hpa-Tg mice depended on gut microbiota. (a) ABX experimental design, WT and Hpa-Tg littermates were put on a course of intragastrically antibiotic cocktail administration for 5 days for gut microbiota depletion After that, 16S rRNA sequencing analysis and AP induction were performed. **(b)** Pancreas (g)/body (g) weight x 1‰. **(c)** Serum amylase. **(d)** Serum lipase. **(e)** Representative images of pancreatic H&E staining. **(f)** Histologic score. **(g)** Serum TNF-α measured by ELISA. **(h)** Serum IL-6 measured by ELISA. **(a-h)** n=5 individuals/group. Data were expressed as mean ± SEM. Differences of data were assessed by assessed by unpaired, two-tailed t test or Mann-Whitney test depending on the sample distribution. Exact *p* levels were all provided. Scale bars, 500 and 200 µm, respectively. ABX, antibiotic cocktail; AP, acute pancreatitis; Cn, caerulein; Hpa-Tg, heparanase-transgenic; rRNA, ribosomal RNA; WT, wild-type.

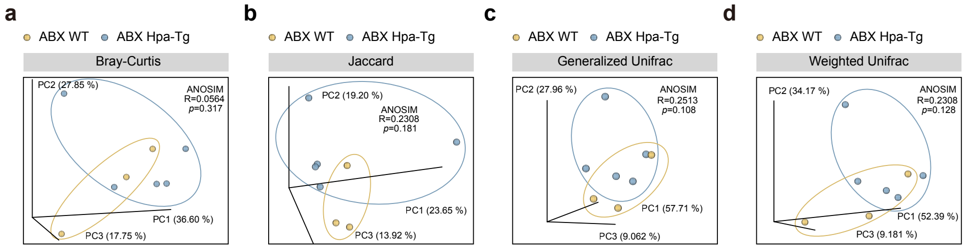

Supplemental Figure 4. Beta-diversity among ABX experimental groups. 16S rRNA sequencing analysis in fecal bacterial DNA from ABX WT mice (n=5) and Hpa-Tg mice (n=3) was performed. PCoA of beta-diversity using Bray-Curtis **(a)**, Jaccard **(b)**, Generalized Unifrac **(c)** and Weighted Unifrac **(d)** metric distance. Differences of data were assessed by ANOSIM test. Exact *p* levels were all provided. ABX, antibiotic cocktail; ANOSIM, analysis of similarities; Hpa-Tg, heparanase-transgenic; PCoA, principal coordinate analysis; rRNA, ribosomal RNA; WT, wild-type.

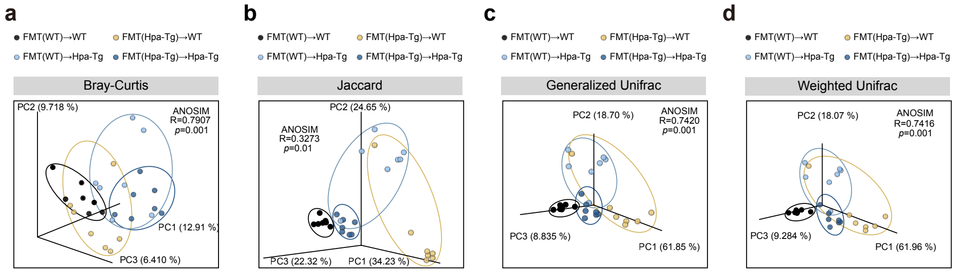

Supplemental Figure 5. Beta-diversity among FMT experimental groups. 16S rRNA sequencing analysis in fecal bacterial DNA from FMT groups was performed. n=7 individuals/group. PCoA of beta-diversity using Bray-Curtis **(a)**, Jaccard **(b)**, Generalized Unifrac **(c)** and Weighted Unifrac **(d)** metric distance. Differences of data were assessed by ANOSIM test. Exact *p* levels were all provided. ANOSIM, analysis of similarities; Hpa-Tg, heparanase-transgenic; PCoA, principal coordinate analysis FMT, fecal microbiota transplantation; rRNA, ribosomal RNA; WT, wild-type.

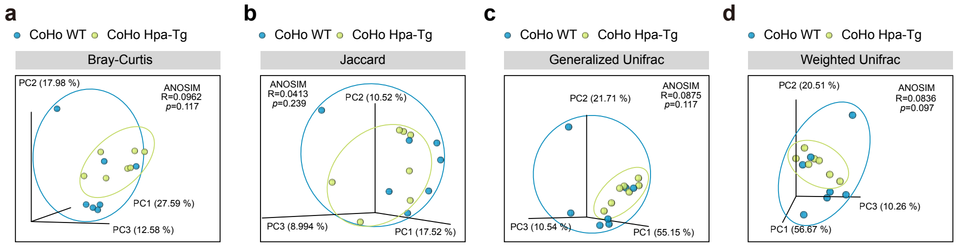

Supplemental Figure 6. Beta-diversity among CoHo WT and CoHo Hpa-Tg groups. 16S rRNA sequencing analysis in fecal bacterial DNA from CoHo WT and CoHo Hpa-Tg groups was performed. n=7 individuals/group. PCoA of beta-diversity using Bray-Curtis **(a)**, Jaccard **(b)**, Generalized Unifrac **(c)** and Weighted Unifrac **(d)** metric distance. Differences of data were assessed by ANOSIM test. Exact *p* levels were all provided. ANOSIM, analysis of similarities; CoHo, cohoused; Hpa-Tg, heparanase-transgenic; PCoA, principal coordinate analysis; rRNA, ribosomal RNA; WT, wild-type.

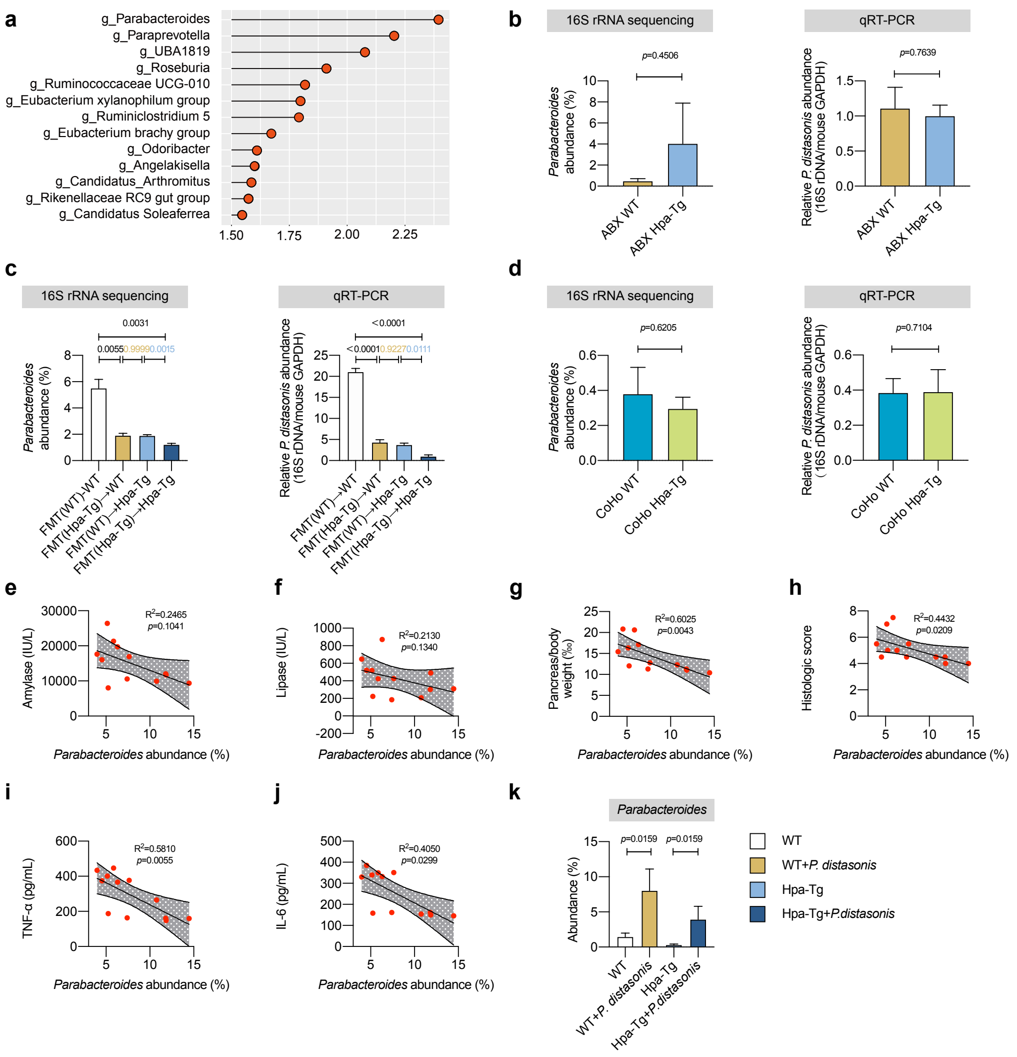

Supplemental Figure 7. *Parabacteroides* was the most important biomarker to distinguish WT and Hpa-Tg mice. (a) VIP score of OPLS-DA. VIP score (calculated based on 16S rRNA sequencing data of Figure 2) was used to rank the ability of different taxa to discriminate between WT and Hpa-Tg mice. A taxon with VIP score >1.5 was considered important in the discrimination. n=15 individuals/group. **(b)** In ABX experiment, *Parabacteroides* abundance was confirmed by 16S rRNA sequencing (n=5/3 in ABX WT/ABX Hpa-Tg group, respectively) and qRT-PCR (using specific primers of *P. distasonis*, n=5 individuals/group). **(c)** In FMT experiment,

Parabacteroides abundance was confirmed by 16S rRNA sequencing and qRT-PCR (using specific primers of *P. distasonis*). n=7 individuals/group. **(d)** In Cohousing experiment, *Parabacteroides* abundance was confirmed by 16S rRNA sequencing and qRT-PCR (using specific primers of *P. distasonis*). n=7 individuals/group. **(e-j)** The correlations between severity indicators of AP and *Parabacteroides* abundance were analyzed using Spearman's correlations. 6 mice each in WT and Hpa-Tg mice were included in the statistics. **(k)** In *Parabacteroides* administration experiment, *Parabacteroides* abundance was confirmed by 16S rRNA sequencing. n=5 individuals/group. Data were expressed as mean ± SEM. Differences of data in two groups were assessed by assessed by unpaired, two-tailed t test or Mann-Whitney test depending on the sample distribution. Differences of data in more than two groups were assessed by ordinary one-way ANOVA or Kruskal-Wallis test depending on the sample distribution. Exact *p* levels were all provided. ABX, antibiotic cocktail; AP, acute pancreatitis; FMT, fecal microbiota transplantation; Hpa-Tg, heparanase-transgenic; OPLS-DA, orthogonal partial least squares discrimination analysis; *P. distasonis*, *Parabacteroides. distasonis*; qRT-PCR, Quantitative RT-PCR; rRNA, ribosomal RNA; VIP, valuable influence on projection; WT, wild-type.

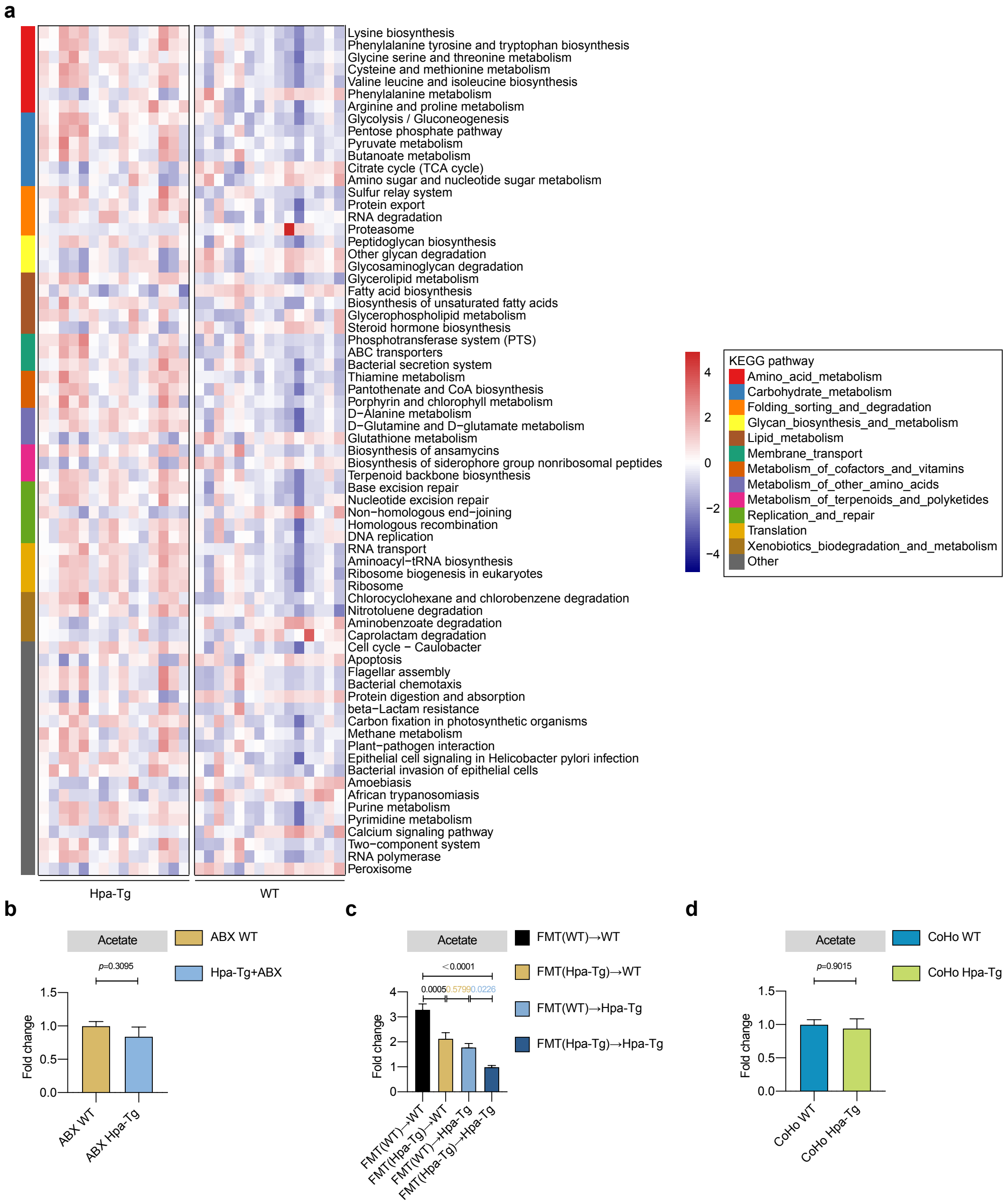

Supplemental Figure 8. Acetate concentration in ABX, FMT and cohousing experiments. (a) The whole annotation of microbial gene function of WT and Hpa-Tg mice on KEGG pathway analysis. Analysis of 16S rRNA sequencing data from Figure 2. n = 15 individuals/group. **(b)** Relative fold change of acetate concentration from cecal content in ABX experiment. n=5 individuals/group. **(c)** Relative fold change of acetate concentration from cecal content in FMT experiment. n=7 individuals/group.

(d) Relative fold change of acetate concentration from cecal content in CoHo WT and CoHo Hpa-Tg mice. n=7 individuals/group. Data were expressed as mean ± SEM. Differences of data in two groups were assessed by assessed by Mann-Whitney test. Differences of data in FMT experiment was assessed by ordinary one-way ANOVA. Exact *p* levels were all provided. ABX, antibiotic cocktail; CoHo, cohoused; FMT, fecal microbiota transplantation; Hpa-Tg, heparanase-transgenic; rRNA, ribosomal RNA; KEGG, kyoto encyclopedia of genes and genomes; WT, wild-type.

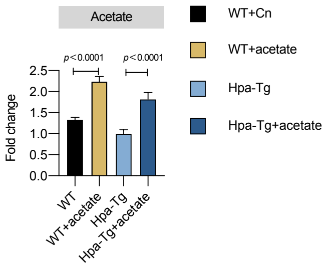

Supplemental Figure 9. Confirmation of acetate enrichment in acetate supplementation experiment. Relative fold change of acetate concentration from cecal content in acetate supplementation experiment. n=9 individuals/group. Data were expressed as mean ± SEM. Differences of data were assessed by ordinary one-way ANOVA. Exact *p* levels were all provided. WT, wild-type; Hpa-Tg, heparanase-transgenic.

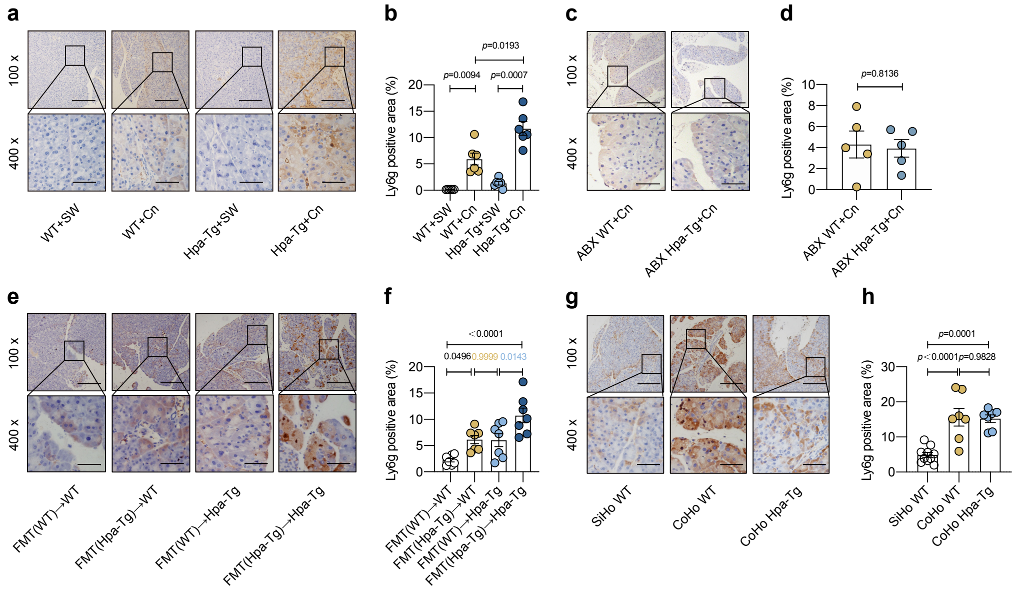

Supplemental Figure 10. Neutrophil infiltration depended on gut microbiota in heparanase-exacerbated acute pancreatitis. WT and Hpa-Tg mice were treated with/without Cn. Representative plots of immunohistochemistry and bar plots of neutrophils in pancreas were shown in **(a)** and **(b)**, respectively. Representative plots of immunohistochemistry and bar plots of neutrophils in pancreas of ABX experiment were shown in **(c)** and **(d)**, respectively. Representative plots of immunohistochemistry and bar plots of neutrophils in pancreas of FMT experiment were shown in **(e)** and **(f)**, respectively. Representative plots of immunohistochemistry and bar plots of neutrophils in pancreas of cohousing experiment were shown in **(g)** and **(h)**, respectively. Differences of data in two groups were assessed by unpaired, two-tailed t test. Differences of data in more than two groups were assessed by ordinary one-way ANOVA. Exact *p* levels were all provided. Scale bars, 200 and 50 µm, respectively. ABX, antibiotic cocktail; FMT, fecal microbiota transplantation; Hpa-Tg, heparanase-

transgenic; Cn, caerulein; CoHo, cohoused. SiHo, housed singly; SW, sterile water; WT, wild-type.

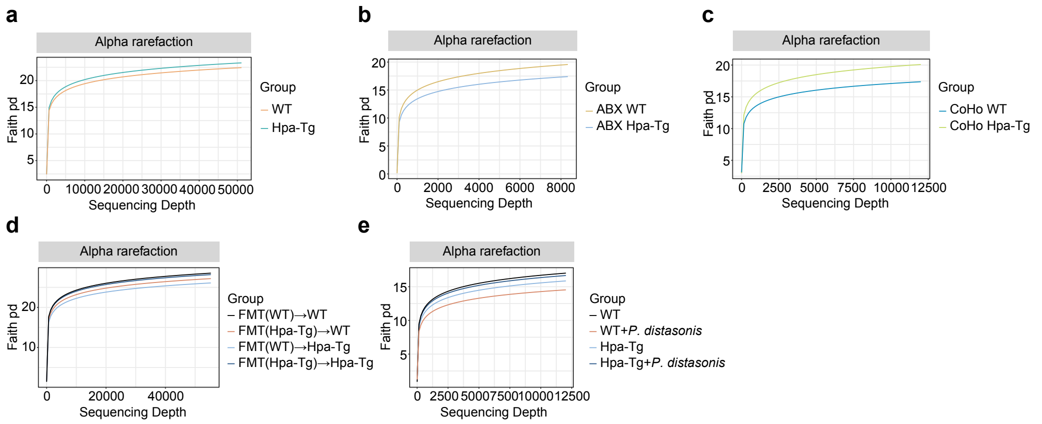

Supplemental Figure 11. Alpha rarefaction curves of all 16S rRNA sequencing analyses. Alpha rarefaction curves (Faith pd) in WT and Hpa-Tg groups **(a)**, ABX experiment **(b)**, CoHo WT and CoHo Hpa-Tg groups **(c)**, FMT experiment **(d)** and the administration of *Parabacteroides* experiment **(e)**. ABX, antibiotic cocktail; CoHo, cohoused; FMT, fecal microbiota transplantation; Hpa-Tg, heparanase-transgenic; *P. distasonis*, *Parabacteroides distasonis*; WT, wild-type.

Supplemental Table 1. Schmidt's score system of pancreatic histopathology.

Pathologic changes	Scores			
	0	1	2	3
Inflammatory infiltrates	Absent	Mild	Moderate	Severe
Edema	Absent	Mild	Moderate	Severe
Parenchymal necrosis	Absent	Mild	Moderate	Severe
Haemorrhage	Absent	Mild	Moderate	Severe

Supplemental Table 2. Primers for qRT-PCR detection (SYBR Green).

Gene name	Forward primer (5'→3')	Reverse primer (5'→3')
β-actin (for mouse)	GGCGGACTGTTACTGAGCTG	CTGCGCAAGTTAGGTTTTGTCA
DEFB1 (for mouse)	GCACAAGAAGGTCACACGGA	CTAAGGTTGCAGATGGGGTGT
CRAMP (for mouse)	CTTCAAGGAACAGGGGGTGG	CTTGAACCGAAAGGGCTGTG
Hpa (for mouse)	ACTTGAAGGTACCGCCTCCG	GAAGCTCTGGAACTCGGCAA
Hpa (for human)	CGGCTAAGATGCTGAAGAGC	TGATGCCATGTAACTGAATCAA

Abbreviations used: CRAMP, cathelicidin-related antimicrobial peptide; DEFB1, β-defensin 1; Hpa, heparanase.

Supplemental Table 3. Primers for qRT-PCR detection (Taqman).

Gene name	Primers (5'→3')	Probe (5'→3')
GAPDH (for mouse)	F: TGCACCACCAACTGCTTAG<br>R: GGATGCAGGGATGATGTTC	CAGAAGACTGTGGATGGCCCT
P. distasonis	F: TGCCTATCAGAGGGGGATAAC<br>R: GCAAATATTCCCATGCGGGAT	CGAAAGTCGGACTAATACCGCATGAAGC

Abbreviations used: *P. distasonis*, *Parabacteroides distasonis.*
